# Emotional indicators of university students: a quasi-experimental pilot study with Hatha-Yoga*

**DOI:** 10.1590/1518-8345.7193.4590

**Published:** 2025-07-28

**Authors:** Vanessa Ferraz Leite, Moisés Kogien, Tatiane Lebre Dias, Margani Cadore Weis Maia, Larissa de Almeida Rézio, Samira Reschetti Marcon

**Affiliations:** 1 Universidade Federal de Mato Grosso, Faculdade de Enfermagem, Cuiabá, MT, Brazil.; 2 Scholarship holder at the Coordenação de Aperfeiçoamento de Pessoal de Nível Superior (CAPES), Brazil.; 3 Universidade Federal de Mato Grosso, Departamento de Educação, Cuiabá, MT, Brazil.

**Keywords:** Yoga, Emotions, Students, Universities, Mental Health, Quasi-Experimental Studies

## Abstract

to analyze the effects of a *Hatha-Yoga* program on the emotional indicators of undergraduate students.

this is a quasi-experimental pilot study carried out with 36 students, with the final sample consisting of 14 in the intervention group and 18 in the control group, totaling 32. The participants’ emotional indicators were assessed before and after the *Hatha-Yoga* intervention, using the Mindful Attention Awareness Scale; Santa Clara Brief Compassion Scale; Self-Compassion Scale; Mini International Neuropsychiatric Interview; Depression, Anxiety, and Stress Scale; Barratt Impulsiveness Scale and Epworth Sleepiness Scale. Descriptive and inferential analyses were carried out using t-tests (paired and for independent groups) to assess the effect of the intervention.

in the intra-group analysis, the intervention group showed an increase in the emotional indicators of compassion, self-compassion and a decrease in anxiety and daytime sleepiness after eight weeks of practicing *Hatha-Yoga*. In the control group, there was a significant decrease in dispositional mindfulness. In the post-test between-groups analysis, moderate effect size differences were found for self-compassion and dispositional mindfulness.

the practice of *Hatha-Yoga* had a positive impact on the students’ emotional indicators, contributing to health promotion and expanded self-care (RBR-10jgv7ky).

## Highlights

**(1)** Positive impact on participants’ emotional indicators. **(2)** Decrease in the effects of the participants’ negative indicators. **(3)** Contribution to health promotion and expanded self-care. **(4)** Reduction in dispositional mindfulness in the absence of yogic practice.

## Introduction

Emotions are internal, involuntary and observable reactions that involve physical manifestations, such as cardiorespiratory changes, hormonal secretions, facial expressions and postural changes, triggered by sensorymotor or remembered events, whose primary role is to maintain life, identify threats or indicate individual or social successes^(1)^. Although innate, emotions can be shaped according to social context and cultural aspects. Emotional states such as joy, sadness, fear, anger, surprise or disgust are considered universal emotions^(2-3)^.

With the progressive complexification of emotional experiences into feelings, and the understanding of these as conscious mental events that simultaneously represent experiences of the body^(4-5)^, and the different conditions that influence its triggers and regulatory perceptions, the university context emerges as an important driver of emotions and feelings, while fostering the expansion of social skills, the acquisition of professional and personal competencies, through new rhythms of study, rules and collective norms^(6-7)^. This mix of emotions and feelings can enhance the development of personal and pro-social skills, but it can also generate suffering and signal maladaptive responses with a progressive emotional worsening disproportionate to certain situations^(8-11)^.

From this perspective, studies show that mindfulness skills, as well as the ability to recognize and express compassion for oneself and others, are directly and indirectly associated with emotional regulation and a better response to mental suffering^(2-2021-2021)^. Furthermore, emotional regulation can be a mechanism for changing the relationship between attentional and compassionate capacities and mental health^(16-18)^.

The World Health Organization (WHO) estimates an increase of more than 25% in the common symptoms of depression and anxiety in the world population since 2020, indicating a global mental health crisis, which underscores the importance of appropriate interventions in the face of intensely stressful situations that require rapid adaptive responses^(19)^.

University students experience high levels of mental distress, exacerbated by the challenging transition from high school to higher education, academic pressures, social and cultural adaptation^(20-22)^. Among these, health students suffer an even greater emotional impact^(23)^ as a result of the overload of personal and academic demands and the role of caring for others, which require high performance, promoting self-blame and competitiveness^(23-25)^. As a result, the combined prevalence of depression and anxiety symptoms among university students reaches 33.6%^(26)^, which is alarming and reflects the urgency of taking a careful look at mental health in this population. In addition, around 14% of university students have outcomes related to suicidal behavior^(27)^ and suffer from sleep quality problems, insomnia and stress, phenomena which, when interconnected, affect their academic performance and mental health^(28)^.

In the search to understand the emotions and feelings experienced by young university students, referred to as emotional indicators (EI), we highlight variables that allow us to assess the subjective and objective reactions associated with basal, primary or universal emotional aspects (affections triggered by the somatosensory pathways linked to the afferent nerves), as well as emotional feelings such as mindfulness, compassion and self-compassion (affections established by the perceptual pathways connected to the efferent nerves^(2-29-30)^.

EI reveal conditions that can have repercussions on serious mental health problems, such as depression, anxiety, chronic stress and sleep disorders, which are often present in health students^(10-31)^. However, they can also act in a healthy way by promoting self-care, self-knowledge and pro-sociability^(32-34)^.

These EI can be regulated by implementing strategies such as pharmacological and non-pharmacological interventions^(35)^. Non-pharmacological interventions, such as physical exercise and body-mind practices, have been shown to be effective in improving the mental health of university students, strengthening pro-social and self-care EIs such as mindfulness, compassion and self-compassion^(36-40)^, as well as providing better adaptive body-mind responses to symptoms of depression, anxiety and stress, impulsivity and daytime sleepiness^(41-43)^.

*Hatha-Yoga* is one of the ancient forms of yoga that reintegrates body, mind and spiritual intuition. Its main objectives are to strengthen the body and stabilize the mind, in order to provide experiences of profound sensory and intuitive transformation^(21-23)^. In contemporary life, *Hatha-Yoga* stands out as a potential tool for addressing students’ emotional and psychological well-being, with the capacity to promote restorative care that integrates body and mind^(44-46)^ and is often strengthened by practices that focus on resilience, mindfulness, compassion and self-compassion^(47-49)^.

*Hatha-Yoga* programs that encourage compassionate experimentation can contribute to the modulation of EI^(31-50-53)^ and can be practiced individually and/or collectively, indoors or outdoors, providing an experience of emotional repair, through autonomic deprogramming and reprogramming of the body that is inseparable from the mind, as well as a global revitalization of life. This revitalization occurs through the creation of new ritualistic realities, with rest and the re-establishment of the body, senses, gestures, mind and spirit^(54-59)^.

In this sense, research has pointed to the psychosomatic benefits of yogic practices for university students^(38-60)^. However, there are few intervention studies with a moderate to high level of evidence that evaluate several EIs simultaneously in this population^(41)^.

In view of the above, the aim of this pilot study is to analyze the effects of a *Hatha-Yoga* program on the emotional indicators of undergraduate students, in order to provide information for planning future confirmatory research, with greater control and sample size. The hypothesis is that exposure to a *Hatha-Yoga* program could regulate emotional indicators such as dispositional mindfulness, compassion and self-compassion, as well as reducing depressive symptoms, anxiety, stress, impulsivity, daytime sleepiness and the risk of suicide among students participating in the intervention.

## Method

### Study design

This is a quasi-experimental, before-and-after pilot study with control group allocation and simple (statistical) masking, based on the Consolidated Standards of Reporting Trials (CONSORT)^(61)^ recommendations for pilot studies.

### Setting

This pilot study was carried out in a covered, large and airy space at a federal public university in a capital city in Brazil’s central-western region. At the time, this space was centrally located in the university, facilitating quick access for the participants, and had the necessary accessories for the *Hatha-Yoga* program.

### Period

The data were collected between May and October 2022.

### Population and selection criteria

In the recruitment phase of the study, the population comprised undergraduate students in Nursing, Medicine, Nutrition and Psychology, with all 806 students regularly enrolled during the data collection period being eligible. Participants under the age of 18, who were doing an internship or who were in the last year or semester of their course, were excluded because they were not on campus during the survey. There were also those who gave incomplete answers to the survey forms and, after that, those who said they were not interested in taking part in the intervention on offer.

In this first phase (recruitment), which took place from May to June 2022, 617 students filled in the survey instruments and became potential subjects for the intervention. Of this total, six participants were excluded due to incomplete questionnaires, totaling 611 valid questionnaires. After this, 311 participants who did not indicate an interest in taking part in the intervention were excluded, making a total of 300 participants eligible to take part in the *Hatha-Yoga* Program.

In addition, psychological counseling and/or yoga or meditation practice, or the use of psychotropic medication, were considered exclusion criteria; however, these did not occur with any of the participants.

### Study variables

This quasi-experimental study used as its primary outcomes emotional indicators assessed before and after applying the *Hatha-Yoga* intervention protocol, namely: dispositional mindfulness, compassion, self-compassion, depressive symptoms, anxious symptoms, stress, current suicide risk, impulsivity and daytime sleepiness. Secondary outcomes used to compare the homogeneity of the intervention and control groups were sociodemographic variables (gender, age, self-reported skin color, sexual orientation, religious practice, marital status, employment status and whether or not they live alone) and academic variables (course enrolled in).

### Study tools

To characterize the sample, we used a sociodemographic questionnaire (gender, age, self-declared skin color, sexual orientation, religious practice, marital status, employment status, housing status) and an academic questionnaire (course) designed for the context of the study.

Seven instruments were used to assess emotional indicators: 1. Mindful Attention Awareness Scale (MAAS), a one-dimensional scale that assesses levels of dispositional mindfulness, made up of 15 items, on a Likert scale from 1 (almost always) to 6 (almost never), with higher scores reflecting higher levels of mindfulness (α = 0.83)^(32)^; 2. Santa Clara Brief Compassion Scale (SCBCS), which aims to assess compassionate behavior towards others, containing five questions, on a Likert scale ranging from 1 (not at all true) to 7 (very true), higher scores indicate greater compassionate behavior (α = 0.84)^(33)^; 3. The Self-Compassion Scale (SCS), to assess self-compassion and relationship with oneself in times of difficulty. It contains 26 questions, distributed in six subscales, with Likert answers, from 1 (almost never) to 5 (almost always), where higher scores indicate greater compassion (α=0.92)^(34)^; 4. Module C of the Mini International Neuropsychiatric Interview (MINI) to assess suicide risk in the last month, consisting of five dichotomous questions (yes/no) that assess suicidal behavior in the last 30 days (four questions) and throughout life (one question). The score for risk stratification can range from 0 to 33 points^(62)^; 5. Depression, Anxiety, and Stress Scale (DASS-21), which has 21 questions and assesses emotional disorder through three subscales: depression (α= 0.92), anxiety (α=0.86) and stress (α=0.90), with a Likert scale response ranging from 0 (not applicable) to 3 (very applicable). The classification of symptoms ranges from normal, mild, moderate, severe and extremely severe^(63)^; 6. Barratt Impulsiveness Scale (BIS-11) to assess three aspects of impulsive behavior. It consists of 30 items on a Likert scale, ranging from 1 (never) to 4 (very often), in which higher scores denote greater impulsiveness (α= 0.62)^(64-65)^; 7. Epworth Sleepiness Scale, which assesses the risk of falling asleep during daily activities. It has items graded from zero (no likelihood of falling asleep) to 3 (strong likelihood of falling asleep), and scores above 10 suggest a diagnosis of excessive daytime sleepiness (α=083)^(66)^.

All of these scales are self-reported and have been adapted for application to adults in the Brazilian context, having shown adequate psychometric properties in their validation studies^(32-34-62-64-66)^.

### Recruitment, sample and allocation for the intervention

The sample power calculation for the test to compare means of independent groups (IG x CG), operated using the G Power software, version 3.1.9.7, considering an average effect size (0.5), significance level of 95% and sample power of 80%, estimated a sample of 128 subjects to be allocated to the two testing groups. However, despite the efforts of the study team, the number was not reached.

It should be noted that, as the minimum sample size was not reached, WhatsApp^®^ and email messages reinforcing the objectives of the study and explaining how the *Hatha-Yoga* program would be carried out were sent as part of the plan to recruit study subjects. However, despite this effort, there was no increase in the number of interested students. Therefore, in order to mitigate hypothesis errors, it was decided to present effect size measures, rather than just p-values.

In the second phase of the study, from June to October 2022, the groups were formed and the eight-week *Hatha-Yoga* program was applied. A randomized and numbered list was drawn from the 300 students eligible for this stage.

Due to the risk of dropouts, the decision was made to send an invitation via e-mail to each of the 300 students who met the eligibility criteria, in order to check their availability to take part in the intervention, according to the times that *Hatha-Yoga* would be offered, with a maximum of three contact attempts. The list followed the logic that if they didn’t accept, the next person was invited. A total of 264 students refused to take part in the intervention stage, due to incompatible schedules, and given the low number of acceptances, there was no randomization. In this phase, called Time Zero, 36 students agreed to take part in the intervention, and the groups were filled by simple 1:1 allocation, organized by a member of the research team (collector), based on the sequence of acceptance, making up two groups, the Intervention Group (IG) (n=18) and the Control Group (CG) (n=18), i.e. the first acceptance was allocated to the IG, the second to the CG and so on, until the list of participants was finalized.

This study opted for an inactive CG (which received the intervention after the end of the study), as inactive conditions make it possible to detect the results of the real intervention, as well as the difficulty of identifying a suitable placebo intervention that potentially had no effect on the emotional indicators assessed^(67)^.

**Figure 1 -:**
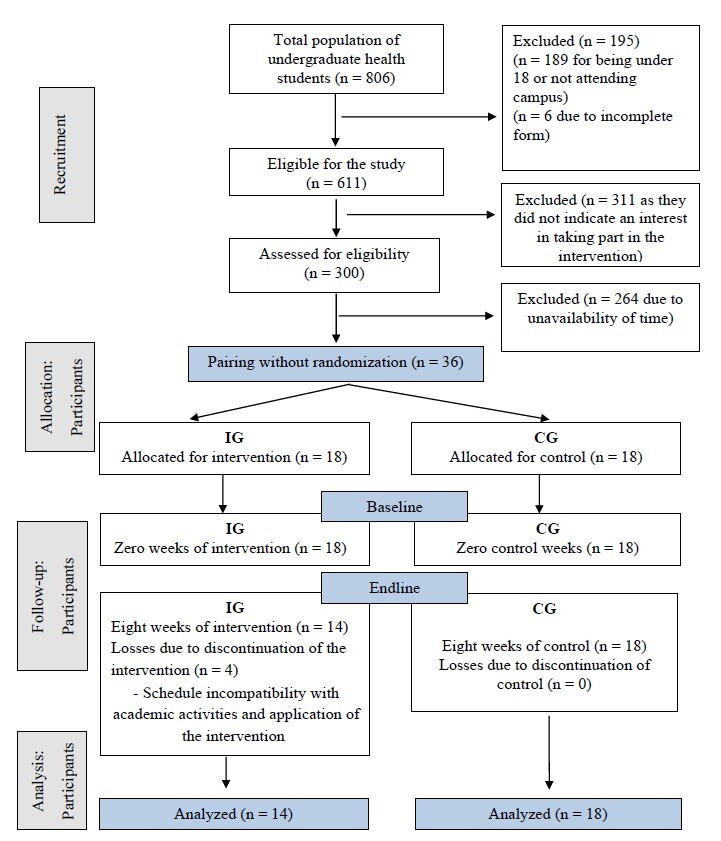
Flowchart of recruitment and allocation of participants based on CONSORT guidelines (2010). Cuiabá, MT, Brazil, 2022

In addition, during the first four weeks of the Program, also known as Time One, there were four dropouts from the IG, resulting in the following allocation at the end of the study: IG (n = 14) and CG (n = 18) (Figure 1).

### Intervention

The intervention in this study consisted of an eight-week *Hatha-Yoga* program. The sessions were taught by the first author, who is a certified *Hatha-Yoga* instructor with improvements in the experiential yoga approach, and who did not take part in the recruitment and evaluation stages of the participants. The program was designed for lay practitioners and yoga beginners, consisting of 16 sessions of 60 minutes of practical class and 15 minutes of theoretical class, totaling 80 minutes, twice a week, from June to October 2022, in a space on the campus of the participating institution.

The *Hatha-Yoga* Program was systematized through a protocol, according to previous guidelines^(68)^, combining techniques inspired by ancient, pre-modern and contemporary *Hatha-Yoga*, such as sacred sounds (*mantras*), stretching, relaxation, physical postures (*asanas*), cleansing practices (*kriyas*), folds (*mudras*), targeted contractions (*bandhas*), breathing practices (*pranayama*), compassionate intention (*sankalpa*), meditation and relaxation, with a total duration of 60 minutes^(45-69-70)^.

During the program, the students were encouraged to attend the sessions in order to continue the research. The IG was subdivided into four schedules throughout the week in order to retain the participants, as well as the possibility of replacement in the event of absence, whenever necessary. The CG participants did not receive the intervention, however, after the end of the research, for ethical reasons, they were invited to a new cycle of the eight-week *Hatha-Yoga* program.

### Data collection

The data were collected mostly in person and took place at two different times: the pre-intervention phase, which took place between May and June 2022, and the post-intervention phase, which took place in October 2022. In the first phase, ten previously trained collectors went to the classrooms and, after authorization from the teacher present, presented the students with details of the study and invited them to take part in the research/intervention. The students who agreed to fill in the questionnaires were presented with the Informed Consent Form (ICF) and instructed to sign it after reading it carefully, indicating their awareness and interest in taking part in the research. Completing the questionnaires in this phase took around 45 minutes.

In the post-intervention phase, after the eight weeks of *Hatha-Yoga* practice with the IG, the same instruments were applied again to the IG participants at the end of the final practice, under the supervision of previously trained collectors, who packed them in sealed envelopes without identifying the groups. At the same time, the CG participants were contacted via e-mail by the collection team and received a Google Drive link with the version of the forms. Only this stage was carried out remotely.

The raw data was double-entered and compared using the Diffchecker^®^ website and stored in Word^®^, Excel^®^ and Google Drive.

### Data analysis

The data was treated descriptively and inferentially. In the descriptive analyses, categorical variables were presented using their absolute and relative frequencies, while continuous variables were presented using their mean scores and respective standard deviations. In inferential analyses, comparisons between proportions were made using the Chi-square test or Fisher’s exact test with Freeman-Halton extension, when necessary. For comparisons of means, t-tests were used for paired samples in intra-group analyses and t-tests for independent samples in analyses between groups. Effect sizes were calculated using the Hedges g-test. The 95% confidence intervals (95%CI) were obtained using bootstrapping procedures (1000 resamples), which provide bias-corrected and accelerated 95%CI. This technique was used to correct for deviations from the normality of the sample distribution and to minimize differences between group sizes^(71)^. The analyses were carried out using the Statistical Package for the Social Sciences (SPSS) software, version 27.

### Ethical aspects

This study was approved by the Research Ethics Committee under opinion no. 49695621.9.0000.8124 and complied with the human research guidelines of Resolution 466/2012 of the National Health Council and the principles of the Declaration of Helsinki. The study was entered in December 2021 in the Brazilian Registry of Clinical Trials (ReBEC) under the registration code RBR-10jgv7ky.

## Results

Thirty-six participants began the intervention proposed in this study. They were equally allocated between IG (n = 18) and CG (n = 18) and were homogeneous (p > 0.05) in terms of sociodemographic and academic characteristics and most of the emotional indicators assessed in this study (Table 1), with the exception of levels of self-compassion, which were higher in CG students (p = 0.005).

The effect of the intervention on the IG participants’ EI (comparison of pre- and post-intervention mean scores) is shown in Table 2.

**Table 1 -:** Sociodemographic and academic characterization and emotional indicators of undergraduate health students according to allocation groups in the pre-intervention period. Cuiabá, MT, Brazil, 2022

**Variables**	**Intervention Group (n** * **= 18)**	Control Group (n* = 18)	**p-value**
	n* (%)	n* (%)	
Gender			
Female	13 (72.2%)	17 (94.4%)	0.177 ^†^
Male	05 (27.8%)	01 (5.6%)	
Age ^‡^	22.06 (±2.31)	23.72 (±7.74)	0.388 (-5.268; 1.449) ^§^
Self-declared skin color			
Non-white	16 (88.9%)	12 (66.7%)	0.228 ^†^
White	02 (11.1%)	06 (33.3%)	
Sexual orientation			
Heterosexual	14 (77.8%)	15 (83.3%)	1.000 ^†^
Minority sexual orientation	04 (22.2%)	03 (16.7%)	
Religious practice			
Yes	08 (44.4%)	12 (66.7%)	0.180
No	10 (55.6%)	06 (33.3%)	
Marital status			
With partner	03 (16.7%)	03 (16.7%)	1.000 ^†^
Without partner	15 (83.3%)	15 (83.3%)	
Employment status			
Works and studies	05 (27.8%)	02 (11.1%)	0.402 ^†^
Only studies	13 (72.2%)	16 (88.9%)	
Housing status			
Lives alone	03 (16.7%)	01 (5.6%)	0.603 ^†^
Does not live alone	15 (83.3%)	17 (94.4%)	
Course			
Nursing	06 (33.3%)	10 (55.6%)	0.342 ^||^
Medicine	01 (5.6%)	02 (11.1%)	
Nutrition	04 (22.2%)	01 (5.6%)	
Psychology	07 (38.9%)	05 (27.8%)	
Mindfulness ^‡^	49.21 (±12.84)	54.17 (±12.07)	0.272 (-14.208; 4.030) ^§^
Compassion ^‡^	25.57 (±7.42)	28.11 (±5.84)	0.287 (-6.343; 2.181) ^§^
Self-compassion ^‡^	68.29 (±12.46)	79.83 (±9.16)	0.005 (-17.877; -3.317) ^§^
Depression ^‡^	15.71 (±6.00)	13.72 (±4.62)	0.297 (-2.574; 4.883) ^§^
Anxiety ^‡^	14.71 (±4.48)	13.44 (±4.69)	0.445 (-2.062; 3.520) ^§^
Stress ^‡^	17.36 (±3.71)	16.83 (±4.73)	0.736 (-2.730; 3.005) ^§^
Suicide risk ^‡^	0.64 (±1.15)	0.44 (±1.29)	0.655 (-0.387; 1.333) ^§^
Impulsivity ^‡^	68.07 (±15.27)	65.89 (±10.90)	0.640 (-6.017; 9.799) ^§^
Excessive sleepiness ^‡^	10.57 (±4.07)	9.44 (±3.85)	0.430 (-2.484; 3.417) ^§^

*n = Sample size; ^†^P value obtained using Fisher’s exact test; ^‡^For the characterization of continuous variables, means and standard deviations were presented; ^§^P value obtained using the t-test for independent samples, with 95%CI obtained using the bootstrapping technique with 1000 resamples; ^||^P value obtained using Fisher’s exact test with Freeman-Halton extension for tables larger than 2x2

**Table 2 -:** Comparison of mean scores for emotional indicators of students allocated to the intervention group at pre- and post-intervention times (n = 14). Cuiabá, Mato Grosso, Brazil, 2022

**Emotional Indicators**	**Intervention Group**					
	**Pre-Intervention (n=14)**	**Post-Intervention (n=14)**	**t**	**p-value**	BCa 95%CI*	**Effect size (g of Hedges)**
	**Mean (SD** ^†^ **)**	**Mean (SD** ^†^ **)**				
Dispositional mindfulness	49.21 (±12.84)	52.29 (±14.96)	1.072	0.288	-8.783; 4.699	0.21
Compassion	25.57 (±7.42)	27.93 (±5.27)	2.168	**0.050**	-4.714; -0.057	0.35
Self-compassion	68.29 (±12.46)	81.14 (±18.86)	3.165	**0.014**	-20.643; -4.084	0.77
Depressive symptoms	15.71 (±6.00)	13.07 (±5.38)	1.459	0.171	-1.286; 6.357	0.43
Anxiety symptoms	14.71 (±4.48)	12.50 (±4.29)	2.302	**0.032**	0.143; 4.348	0.47
Stress	17.36 (±3.71)	16.57 (±2.34)	0.738	0.507	-1.148; 2.863	0.24
Current suicide risk	0.64 (±1.15)	1.64 (±5.11)	0.717	0.550	-3.929; 0.643	0.30
Impulsivity	68.07 (±15.27)	66.43 (±16.48)	0.733	0.500	-1.929; 5.237	0.10
Daytime sleepiness	10.57 (±4.07)	9.21 (±3.91)	2.464	**0.045**	0.357; 2.400	0.32

*BCa 95%CI = Bias-corrected and accelerated 95% confidence interval; ^†^SD = Standard deviation

There was an increase in levels of compassion (p<0.050) and self-compassion (p<0.014), as well as a reduction in anxiety symptoms (p<0.032) and daytime sleepiness (p<0.045) after completing the eight-week *Hatha-Yoga* program.

In the same period, the participants allocated to the group that did not receive the intervention (CG) showed a decrease in the emotional indicator of dispositional mindfulness (p<0.013), with no evidence of improvement or worsening in the other EIs assessed (Table 3).

**Table 3 -:** Comparison of mean scores for emotional indicators of students allocated to the control group at pre- and post-test times (n = 18). Cuiabá, MT, Brazil, 2022

**Emotional indicators**	**Control Group**					
	**Pre-test (n=18)**	**Post-test (n=18)**	**t**	**p-value**	BCa 95%CI*	**Effect size (g of Hedges)**
	**Mean (SD** ^†^ **)**	**Mean (SD** ^†^ **)**				
Dispositional mindfulness	54.17 (±12.07)	43.72 (±12.87)	3.235	**0.013**	3.778; 16.919	0.80
Compassion	28.11 (±5.84)	27.00 (±8.36)	0.814	0.427	-1.426; 3.722	0.15
Self-compassion	79.83 (±9.16)	73.61 (±13.83)	0.218	0.134	-6.121; 4.255	0.52
Depressive symptoms	13.72 (±4.62)	14.56 (±5.78)	0.753	0.440	-7.167; 0.056	0.15
Anxiety symptoms	13.44 (±4.69)	11.94 (±4.56)	1.205	0.281	-2.778; 1.116	0.31
Stress	16.83 (±4.73)	14.33 (±5.16)	1.676	0.138	-0.444; 3.556	0.48
Current suicide risk	0.44 (±1.29)	3.33(±8.64)	1.401	0.279	-0.222; 5.526	0.55
Impulsivity	65.89 (±10.90)	68.11 (±11.41)	1.481	0.193	-5.608; 0.667	0.19
Daytime sleepiness	9.44 (±3.85)	10.94 (±4.54)	1.448	0.165	-3.489; 0.500	0.34

*BCa 95%CI = Bias-corrected and accelerated 95% confidence interval; ^†^SD = Standard deviation

Finally, Table 4 compares the mean EI scores of students allocated to the IG and CG post-test and no statistically significant differences were observed in these parameters, however, the differences between the mean scores of the self-compassion and dispositional mindfulness levels stand out, which showed the largest effect sizes, respectively g = 0.66 and g = 0.60, consistent with moderate effect sizes.

**Table 4 -:** Comparison of mean scores for emotional indicators of students allocated to the intervention group (n = 14) and the control group (n = 18) at the post-test moment. Cuiabá, MT, Brazil, 2022

**Emotional indicators**	**Post-test**					
	**Intervention Group (n = 14)**	**Control Group (n = 18)**	**t**	**p-value**	BCa 95%CI*	**Effect size (g of Hedges)**
	**Mean (SD** ^†^ **)**	**Mean (SD** ^†^ **)**				
Dispositional mindfulness	52.29(±14.96)	43.72 (±12.87)	1.739	0.089	-1.280; 19.238	0.60
Compassion	27.93(±5.27)	27.00 (±8.36)	0.363	0.702	-3.850; 6.249	0.13
Self-compassion	81.14(±18.86)	73.61 (±13.83)	1.575	0.104	-2.297; 18.024	0.66
Depressive symptoms	13.07(±5.38)	14.56 (±5.78)	0.742	0.522	-5.287; 2.696	0.26
Anxiety symptoms	12.50(±4.29)	11.94 (±4.56)	0.351	0.464	-2.555; 3.999	0.12
Stress	16.57 (±2.34)	14.33 (±5.16)	1.504	0.728	-0.581; 5.129	0.52
Current suicide risk	1.64 (±5.11)	3.33(±8.64)	0.648	0.143	-6.413; 2.631	0.22
Impulsivity	66.43 (±16.48)	68.11 (±11.41)	0.341	0.735	-9.901; 6.092	0.12
Daytime sleepiness	9.21(±3.91)	10.94 (±4.54)	1.135	0.266	-4.648; 1.167	0.39

*BCa 95%CI = Bias-corrected and accelerated 95% confidence interval; ^†^SD = Standard deviation

## Discussion

This pilot study analyzed whether emotional indicators of university students could be altered after an eight-week *Hatha-Yoga* program. Despite the absence of statistically significant differences between the IG and CG in the post-test period, there was evidence of improvements in some EIs among the students who took part in the intervention. These findings are congruent with previous research that has shown yogic practice to be potentially beneficial for emotional regulation^(14)^, the balance of emotional reactivity and derogatory feelings/affections^(53-63-72-73)^, since body-mind practices that cultivate compassion or loving kindness contribute to the maintenance, expansion and improvement of positive emotions and the strengthening of favorable interpersonal/pro-social behaviors^(53-64-74-75)^.

In this study, the practice of *Hatha-Yoga* followed an intervention protocol that integrated yogic psychophysical techniques and a moment of welcoming the individuals for theoretical-educational exchanges on the social ethics of yoga (*yamas*) and self-observances (*niyamas*), as well as sharing experiences on expanded, collective and affective self-care^(23-24-76-77)^. The practice was applied twice a week for eight weeks and contributed to increasing the students’ capacity for compassion and self-compassion, while also reducing the severity of anxiety symptoms and daytime sleepiness in this group.

Compassion, in general, can stimulate calm, affective contact and affiliation^(49-78-79)^ and, in the long term, can link life purposes, feelings of closeness, positive affections and collective connection behaviors^(75)^. Since compassion can be modulated, interventions that stimulate it produce physical and mental intrapersonal and interpersonal benefits, as well as cooperation and tolerance towards the suffering of others^(80-82)^, which are fundamental aspects for health professionals in training^(38)^. Therefore, practices that cultivate compassion can be a means and an ally for alleviating suffering and promoting individual and social flourishing^(48-83-86)^.

Self-compassion is the ability to consciously have compassion for oneself, and kindness in symbiosis with shared humanity and mindfulness^(87-88)^. Self-compassion not only awakens intrapersonal coping skills, but can also raise awareness of greater acceptance and the search for social support in times of challenge and emotional suffering^(81-89)^.

The increase in self-compassion levels found in the IG in this study is in line with the results found in the literature on body-mind practices, which show that these can contribute positively to modulating self-compassion^(87-90)^. In addition, similar results were observed in a randomized experimental study with 100 Indian nursing students, using the same type and period of intervention. It found that yoga was effective in improving self-compassion and mindfulness when comparing groups (intervention and control)^(60)^. Another quasi-experimental study with American undergraduate nursing students (n=73), aimed at promoting self-compassion, offered weekly yoga classes (one hour) for 12 weeks and found that the IG showed greater cultivation of kindness/self-kindness, one of the primary facets of self-compassion, than the CG^(40)^. In this study, no statistically significant differences were found between the levels of self-compassion between IG and CG in the post-test, however, the effect size of the difference in means was moderate, which, in line with the findings above, may indicate an important effect of *Hatha-Yoga* practice on this SI, which was not evidenced due to the small sample size of this study.

Increasing levels of compassion and self-compassion strengthens the training process of university students beyond the acquisition of clinical and managerial skills, as it enhances co-responsibility and autonomy for self-care and, above all, motivates the formation of support networks, such as friends, colleagues, teachers, family members, support groups, among others^(13-91-92)^. Self-compassion makes it possible to take care of oneself in an expanded way, since it creates diverse socio-material networks, strengthening fundamental bonds for professionals directly involved in human care. Caring for others implies caring for oneself, and support networks are fundamental elements in this process^(76-77-92)^.

With regard to anxious symptoms, an EI that decreased in the IG after practicing *Hatha-Yoga*, these are mental and subjective phenomena or come from bodily experiences stimulated and distortedly learned in chaotic contemporary daily life. These symptoms trigger the alert system in the brain, via the hypothalamic-pituitary-adrenal (HPA) axis^(49-93-94)^, which activates the “fight and flight” mechanism. This mechanism, which used to be triggered sporadically in a state of homeostasis, becomes permanently activated when the HPA is activated, overloading the individual with reactivity and derogatory subjectivities, inattention, irritability and impulsiveness which, in most cases, are disproportionate to reality^(93)^. *Hatha-Yoga* interventions applied to university students reaffirm the practice’s potential to mitigate the severity of anxiety symptoms^(95)^.

With regard to daytime sleepiness, although this is not an emotion in the exact sense, its presence can have a negative effect on quality of life and be an indication of cognitive, behavioral, mental and emotional irregularity for university students^(30-96-97)^. Daytime sleepiness can be conceptualized as an individual’s reduced ability to remain awake and alert during normal daytime hours, resulting in naps and lapses of sleepiness or sleep^(98-99)^.

For the yogic understanding, the sufferings (*duhkha-traya*) of sleep are psychophysical incongruities (*viksepa*) arising from the individual’s wrong perceptions (*bhrantidarsana*) of internal and external stimuli (*klesas*) and their difficulty in maintaining homeostasis between body and mind (*manas*)^(100)^. Regardless of how the phenomenon is viewed, research shows that daytime sleepiness can significantly harm the mental health of university students in the process of academic training^(101-102)^.

A systematic review and meta-analysis on sleep quality and insomnia found that practices such as yoga can reduce daytime sleepiness in adults^(103)^. Another systematic review on clinical applications of experimental studies with meditative practices based on mindfulness, yoga and transcendental meditation, showed a positive outcome in improving sleep quality in young people and adults^(104)^. Bodily-minded interventions contribute to neuronal synchronization and, consequently, to improving the basic global functions of practitioners^(7-49)^. In this way, yogic meditation can play a regulatory role not only in cognitive performance, but also in promoting an integral bodily-sensory-mental restoration^(104)^.

An important finding of the study refers to the dispositional mindfulness indicator (DMI), which although it remained stable in the IG in the pre- and post-test comparison, decreased significantly in the CG in the same period. In addition, despite the lack of statistical significance in the analysis between the intervention and control groups, the effect size found in the post-test was moderate, indicating an important difference between the values of the mean scores analyzed, which could be clinically relevant. One possibility for this is that the intervention group may have benefited from practicing *Hatha-Yoga* and, even though it didn’t contribute to an increase in DMI levels, it prevented a decrease in DMI, as shown in the CG.

The attentional process comprises a cycle in which the individual constantly alternates between attention, digression, awareness of the digression and return to focus^(105-106)^. In this process, wandering can gradually be reduced as attentional experiences are cultivated through meditative practices^(49-82-107-108)^.

Although there are still few scientific publications on yoga interventions for comparative assessment of DMI in university students, the available evidence suggests improvements in DMI levels following the implementation of yogic interventions, probably due to improved concentration and a reduction in mental wandering resulting from daily practices^(90-109-110)^.

It is important to note that DMI is present in the cultivation of compassion and self-compassion, and studies have increasingly pointed to positive correlations between these and dispositional attentional capacity^(29-79-108)^, as well as contributing to the emotional regulation of anxious symptoms^(111)^ and modulating sleep quality^(41)^.

This study is not without its limitations. It is worth noting that the simple allocation of participants by order of acceptance in the constitution of the control and experimental groups was used, as this may have made it difficult to measure the real effects of the intervention on the emotional indicators assessed. In addition, there was a lack of follow-up (attrition) from the participants during the application phase of the eight-week *Hatha-Yoga* protocol, even though retention strategies had been adopted, such as flexible practice times and sending weekly reminders and messages of encouragement to participants in the intervention group. Attrition is a unique challenge in longitudinal evaluations and can significantly compromise the validity and integrity of an investigation. New randomized studies are suggested, with a larger sample size to compensate for loss to follow-up and which follow students throughout their training process and include students other than those in the health field.

Another important limitation is the sample size recruited, which fell short of the minimum stipulated in the sample calculation, which may have contributed to the fact that possible effects of the intervention on emotional indicators were not detected. Therefore, to mitigate this limitation, the authors chose to present and discuss the results using effect size measures that demonstrate how strong the study’s findings are. Finally, the adoption of an inactive control group can be seen as a limitation, and it is suggested that future studies could include an active control group in order to provide comparisons of the effects of practicing *Hatha-Yoga* with other integrative and complementary approaches.

As a contribution, the study advances scientific knowledge by proposing a non-pharmacological intervention as a form of mental health care for students in the university context, with significant results in the modulation of EI, which, when balanced, have repercussions on better academic performance and better adaptive responses to the demands of everyday university life. In addition, *Hatha-Yoga* is an integrative practice that strengthens individuals in the personal and interpersonal/pro-social spheres, making it safer and more responsible when based on scientific evidence.

Finally, it should be emphasized that the results should be interpreted with caution because, as a pilot study, they provide input for planning future research, rather than constituting a definitive conclusion.

## Conclusion

This study aimed to preliminarily evaluate the EI of undergraduate health students pre- and post-intervention with an eight-week *Hatha-Yoga* program, resulting in a significant increase in the capacity for compassion, self-compassion, as well as a reduction in anxious symptoms and daytime sleepiness in the intervention group, after eight weeks of applying a *Hatha-Yoga* program. In the control group there was a significant decrease in DMI levels. Although no statistically significant differences were found in the SI of the IG and CG in the post-test, the differences between the means of self-compassion and DMI between these two groups had a moderate effect size, which may suggest clinically relevant differences.

From this perspective, higher education institutions should consider the development of non-pharmacological, restorative interventions such as integrative and complementary health practices, especially those of the corporeal-sensory-mental type such as *Hatha-Yoga*, which incorporates the motivation of recognition and integrated cultivation of mindfulness, capacity for compassion and self-compassion, to support university students’ expanded self-care. Future studies that include a larger sample population and long-term follow-up are needed to ensure a beneficial effect and its repercussions.
