# Supplementary material for: Emotional indicators of university students: a quasi-experimental pilot study with Hatha-Yoga
Source: Rev Lat Am Enfermagem. 2025 Jul 28;33:e4590. doi: 10.1590/1518-8345.7193.4590 (PMC12306933; doi:10.1590/1518-8345.7193.4590)
Supplement: Translation

# Translation: Indicadores emocionais de estudantes universitários: um estudo-piloto quasi-experimental com *Hatha-Yoga* *

**DOI:** 10.1590/1518-8345.7193.4591

**Authors:** Vanessa Ferraz Leite, Moisés Kogien, Tatiane Lebre Dias, Margani Cadore Weis Maia, Larissa de Almeida Rézio, Samira Reschetti Marcon

**Affiliations:** 1 Universidade Federal de Mato Grosso, Faculdade de Enfermagem, Cuiabá, MT, Brasil.; 2 Bolsista da Coordenação de Aperfeiçoamento de Pessoal de Nível Superior (CAPES), Brasil.; 3 Universidade Federal de Mato Grosso, Departamento de Educação, Cuiabá, MT, Brasil.

**Keywords:** Yoga, Emoções, Estudantes, Universidades, Saúde Mental, Estudos Quasi-Experimentais

## Abstract

analisar os efeitos de um programa de *Hatha-Yoga* nos indicadores emocionais de estudantes de graduação.

estudo-piloto quasi-experimental, desenvolvido com 36 estudantes, sendo a amostra final composta por 14 no grupo intervenção e 18 no grupo controle, totalizando 32. Os indicadores emocionais dos participantes foram avaliados pré e pós-intervenção de *Hatha-Yoga,* por meio da *Mindful Attention Awareness Scale*; *Santa Clara Brief Compassion Scale*; *Self-Compassion Scale*; *Mini International Neuropsychiatric Interview*; *Depression, Anxiety, and Stress Scale*; *Barratt Impulsiveness Scale* e *Epworth Sleepiness Scale*. Realizado análises descritivas, inferenciais com testes t (pareados e para grupos independentes) para avaliação do efeito da intervenção.

nas análises intragrupos, verificou-se, no grupo intervenção, aumento nos indicadores emocionais de compaixão, autocompaixão e diminuição de ansiedade e sonolência diurna após oito semanas de prática de *Hatha-Yoga*. No grupo controle, houve decréscimo significativo da atenção plena disposicional. Na análise entre grupos pós-teste encontrou-se diferenças de tamanhos de efeito moderados para autocompaixão e atenção plena disposicional.

a prática de *Hatha-Yoga* impactou positivamente nos indicadores emocionais dos estudantes, contribuindo para a promoção de saúde e o cuidado de si ampliado (RBR-10jgv7ky).

## Destaques

(1) Impacto positivo nos indicadores emocionais dos participantes. (2) Diminuição dos efeitos dos indicadores negativos dos participantes. (3) Contribuição para a promoção de saúde e o cuidado de si ampliado. (4) Redução da atenção plena disposicional na ausência da prática yóguica.

## Introdução

As emoções são reações internas, involuntárias e observáveis, que envolvem manifestações físicas, como alterações cardiorrespiratórias, secreções hormonais, expressões faciais e mudanças posturais, desencadeadas por eventos sensório-motores ou rememorados, cujo papel primordial é manter a vida, identificar ameaças ou indicar êxitos individuais ou sociais^(1)^. Embora inatas, as emoções podem ser moldadas conforme o contexto social e aspectos culturais. Estados emocionais como alegria, tristeza, medo, raiva, surpresa ou repugnância são considerados emoções universais^(2-3)^.

Com a progressiva complexificação das experiências emocionais para sentimentos e a compreensão destes como eventos mentais conscientes que representam simultaneamente experiências do corpo^(4-5)^, e as diferentes condições que influenciam nos seus desencadeamentos e percepções regulatórias, o contexto universitário desponta como um importante propulsor das emoções e dos sentimentos, enquanto propicia a ampliação de habilidades sociais, de aquisição de competências profissionais e pessoais, mediante novos ritmos de estudo, de regras e normas coletivas^(6-7)^. Esse misto de emoções e sentimentos pode potencializar o desenvolvimento de habilidades pessoais e pró-sociais, mas também pode gerar sofrimento e sinalizar respostas desadaptativas com um progressivo agravamento emocional desproporcional a determinadas situações^(8-11)^.

Diante dessa perspectiva, estudos apontam que a capacidade de atenção plena, bem como habilidades de reconhecer e expressar compaixão por si e pelos outros, estão direta e indiretamente associadas à regulação emocional e à melhor resposta a sofrimentos mentais^(2-12-15)^. Além disso, a regulação emocional pode ser um mecanismo de mudança na relação entre capacidades atencionais, compassivas e saúde mental^(16-18)^.

A Organização Mundial de Saúde (OMS) estima um aumento de mais de 25% nos sintomas comuns de depressão e ansiedade na população mundial desde 2020, indicando uma crise de saúde mental global, que ressalta a importância de intervenções adequadas diante de situações intensamente estressantes e que exigem rápidas respostas adaptativas^(19)^.

Estudantes universitários vivenciam altos níveis de sofrimento mental, exacerbados pela desafiadora transição do ensino médio para o ensino superior, pressões acadêmicas, adaptação social e cultural^(20-22)^. Entre esses, os estudantes da área da saúde sofrem um impacto emocional ainda maior^(23)^ em decorrência da sobrecarga de demandas pessoais, acadêmicas e da atuação no cuidado com o outro, que exigem alto desempenho, promovendo autocobrança e competitividade^(23-25)^. Consequentemente, a prevalência combinada de sintomas de depressão e ansiedade entre estudantes universitários atinge 33,6%^(26)^, o que demonstra uma situação alarmante, refletindo a urgência de um olhar cuidadoso sobre a saúde mental nesta população. Além disso, cerca de 14% dos universitários apresentam desfechos relacionados ao comportamento suicida^(27)^ e sofrem com problemas na qualidade do sono, insônia e estresse, fenômenos que, interligados, afetam seu desempenho acadêmico e sua saúde mental^(28)^.

Na busca pela compreensão das emoções e sentimentos vivenciados pelos jovens universitários, referidos como indicadores emocionais (IE), destacam-se variáveis que permitem avaliar as reações subjetivas e objetivas associadas aos aspectos emocionais basais, primários ou universais (afecções acionadas pelas vias somatossensoriais vinculadas aos nervos aferentes), bem como sentimentos emocionais, como atenção plena, compaixão e autocompaixão (afetos estabelecidos pelas vias perceptivas conectadas aos nervos eferentes^(2-29-30)^.

Os IE evidenciam condições que podem repercutir em problemas sérios de saúde mental, como depressão, ansiedade, estresse crônico e alterações do sono, frequentemente presentes em estudantes da área da saúde^(10-31)^. No entanto, também podem atuar salutarmente favorecendo o autocuidado, autoconhecimento e pró-sociabilidade^(32-34)^.

Esses IE podem ser regulados por meio da implementação de estratégias, como as intervenções farmacológicas e as não farmacológicas^(35)^. As intervenções não farmacológicas, como exercícios físicos e práticas corpo-mente, têm demonstrado eficácia na melhoria da saúde mental dos estudantes universitários, fortalecendo IE pró-sociais e de autocuidado como a atenção plena, compaixão e a autocompaixão^(36-40)^, bem como proporcionando melhores respostas adaptativas corpo-mente aos sintomas de depressão, ansiedade e estresse, impulsividade e sonolência diurna^(41-43)^.

O *Hatha-Yoga* é uma das formas de *yoga* antiga que reintegra corpo, mente e a intuição espiritual. Seus principais objetivos são fortalecimento corporal, estabilização mental, para oportunizar experiências de uma profunda transformação sensitiva e intuitiva^(21-23)^. Na vida contemporânea, o *Hatha-Yoga* se destaca como uma ferramenta potencial para abordar o bem-estar emocional e psicológico dos estudantes, com capacidade para promover um cuidado restaurativo que integra corpo e mente^(44-46)^ e, muitas vezes, fortalecido por práticas que enfocam resiliência, atenção plena, compaixão e autocompaixão^(47-49)^.

Programas de *Hatha-Yoga* que incentivam experimentações compassivas podem contribuir para a modulação dos IE^(31-50-53)^ e podem ser praticados de forma individual e/ou coletiva, em ambientes internos ou externos, proporcionando uma experiência de reparação emocional, por meio de uma desprogramação e reprogramação autonômica do corpo indissociável da mente, além da revitalização global da vida. Essa revitalização ocorre a partir da criação de novas realidades ritualísticas, com o descanso e o reestabelecimento corporal, sensorial, gestual, mental e espiritual^(54-59)^.

Nesse sentido, pesquisas apontam benefícios psicossomáticos de práticas yóguicas para estudantes universitários^(38-60)^. Contudo, são escassos os estudos de intervenção de moderado a alto nível de evidência que avaliam diversos IE simultaneamente nessa população^(41)^.

Diante do exposto, como um estudo-piloto, objetiva-se analisar os efeitos de um programa de *Hatha-Yoga* nos indicadores emocionais de estudantes de graduação, visando fornecer informações para o planejamento de pesquisas confirmatórias futuras, com maior controle e tamanho amostral. Parte-se da hipótese de que a exposição a um programa de *Hatha-Yoga* poderia regular indicadores emocionais como atenção plena disposicional, compaixão e autocompaixão, além de reduzir sintomas depressivos, ansiosos, de estresse, impulsividade, sonolência diurna e o risco de suicídio entre estudantes participantes da intervenção.

## Método

### Delineamento do estudo

Trata-se de um estudo-piloto quasi-experimental, do tipo antes e depois, com alocação de grupo controle e mascaramento simples (estatístico), baseado nas recomendações do *Consolidated Standards of Reporting Trials* (CONSORT)^(61)^ para estudos-piloto.

### Cenário

Este estudo-piloto foi conduzido em um espaço coberto, amplo e arejado de uma Universidade pública federal de uma capital da região Centro-Oeste brasileira. O referido espaço, no período, ficava centralizado na universidade, facilitando o acesso rápido dos participantes e contava com os acessórios necessários para a execução do Programa de *Hatha-Yoga*.

### Período

Os dados foram coletados entre maio e outubro de 2022.

### População e critérios de seleção

Na fase de recrutamento do estudo, a população compreendeu estudantes universitários de cursos de graduação em Enfermagem, Medicina, Nutrição e Psicologia, sendo elegíveis todos os 806 alunos regularmente matriculados no período de coleta de dados. Foram excluídos os participantes com idade inferior a 18 anos completos, que cursavam estágio, internato ou que estavam no último ano ou semestre do curso matriculado, por não frequentarem o *campus* durante a realização da pesquisa. Ainda, os que responderam incompletamente aos formulários da pesquisa e, após isso, os que informaram não ter interesse em participar da intervenção ofertada.

Na primeira fase (recrutamento), que ocorreu de maio a junho de 2022, 617 estudantes preencheram os instrumentos de pesquisa e se tornaram potenciais sujeitos para a intervenção. Do total, excluíram-se seis participantes por questionários incompletos, totalizando 611 questionários válidos. Após isso, foram excluídos 311 participantes que não indicaram interesse em participar da intervenção, totalizando, portanto, 300 participantes elegíveis para ingressar no Programa de *Hatha-Yoga.*

Ainda, a realização de acompanhamento psicológico e/ou prática do tipo *yoga* ou meditação ou uso de medicação psicotrópica foram considerados critérios de exclusão, no entanto, tais aspectos não ocorreram com nenhum dos participantes.

### Variáveis do estudo

Este estudo quasi-experimental adotou como desfechos primários indicadores emocionais avaliados pré e pós aplicação do protocolo de intervenção com *Hatha-Yoga*, sendo eles: atenção plena disposicional, compaixão, autocompaixão, sintomas depressivos, sintomas ansiosos, estresse, risco de suicídio atual, impulsividade e sonolência diurna. Como desfechos secundários, utilizados para comparação da homogeneidade dos grupos intervenção e controle, utilizaram-se variáveis sociodemográficas (sexo, idade, cor da pele autodeclarada, orientação sexual, prática religiosa, status conjugal, status laboral e se mora sozinho ou não) e acadêmica (curso matriculado).

### Instrumentos de estudo

Para caracterização amostral utilizou-se questionário sociodemográfico (sexo, idade, cor da pele autodeclarada, orientação sexual, prática religiosa, situação conjugal, status conjugal, status laboral, status de moradia) e acadêmico (curso) elaborado para o contexto do estudo.

Para avaliação de indicadores emocionais utilizou-se 7 instrumentos: 1. *Mindful Attention Awareness Scale* (MAAS), escala unidimensional, que avalia os níveis de atenção plena disposicional, composta por 15 itens, em escala Likert de 1 (quase sempre) a 6 (quase nunca), pontuações mais altas refletem níveis mais altos de atenção plena (α = 0,83)^(32)^; 2. *Santa Clara Brief Compassion Scale* (SCBCS), que objetiva avaliar comportamentos de compaixão a terceiros, contendo cinco questões, em escala Likert que variam de 1 (nada verdadeiro) a 7 (muito verdadeiro), escores mais altos indicam maior comportamento de compaixão (α = 0,84)^(33)^; 3. *Self-Compassion Scale* (SCS), para avaliação da autocompaixão e relacionamento consigo mesmo, em momentos de dificuldade. Contém 26 questões, distribuídas em seis subescalas, com respostas em Likert, de 1 (quase nunca) a 5 (quase sempre), onde maiores pontuações indicam maior compaixão (α=0,92)^(34)^; 4. Módulo C da *Mini International Neuropsychiatric Interview* (MINI) para avaliação do risco de suicídio no último mês, constituído por cinco perguntas dicotômicas (sim/não) que avaliam comportamentos suicidas nos últimos 30 dias (quatro perguntas) e ao longo da vida (uma pergunta). O escore para estratificação do risco pode variar de 0 a 33 pontos^(62)^; 5. *Depression, Anxiety, and Stress Scale* (DASS-21), o qual apresenta 21 questões e avalia a desordem emocional por meio de três subescalas: depressão (α= 0,92), ansiedade (α= 0,86) e estresse (α=0,90), com resposta em escala de Likert, variando de 0 (não se aplicou) a 3 (aplicou-se muito). A classificação dos sintomas compreende níveis entre normal, suave, moderado, severo e extremamente severo^(63)^; 6. *Barratt Impulsiveness Scale* (BIS-11) para avaliação de três aspectos do comportamento impulsivo. Constituída por 30 itens, em escala de Likert, entre 1 (nunca) a 4 (muito frequentemente), na qual maiores escores denotam maior impulsividade (α= 0,62)^(64-65)^; 7. *Epworth Sleepiness Scale,* que avalia o risco de adormecer durante as atividades cotidianas. Apresenta itens graduados de zero (nenhuma probabilidade de pegar no sono), 3 (forte probabilidade de pegar no sono), e escores acima de 10 sugerem diagnóstico de sonolência diurna excessiva (α=083)^(66)^.

Todas essas escalas são autorrelatadas e sofreram adaptações para aplicação em adultos no contexto brasileiro, tendo apresentado adequadas propriedades psicométricas em seus estudos de validação^(32-34-62-64-66)^.

### Recrutamento, amostra e alocação para a intervenção

O cálculo de poder amostral para teste de comparação de médias de grupos independentes (GI x GC), operacionalizado por meio do *software G Power*, versão 3.1.9.7, considerando tamanho de efeito médio (0,5), nível de significância de 95% e poder amostral de 80%, estimou amostra de 128 sujeitos a serem alocados nos dois grupos de testagem. Entretanto, apesar dos esforços da equipe do estudo, o quantitativo não foi atingido.

Destaca-se que, ao não se atingir o tamanho amostra mínimo previsto, mensagens de *WhatsApp* ^®^ e via *e-mail* foram encaminhadas reforçando os objetivos do estudo e explicando como o programa de *Hatha-Yoga* seria realizado, como parte do plano de recrutamento de sujeitos de estudo. Entretanto, apesar do esforço, não houve aumento no número de estudantes interessados. Assim, para mitigar erros de hipóteses, optou-se por apresentar medidas de tamanho de efeitos, ao invés de apresentar apenas valores de p.

Na segunda fase do estudo, de junho a outubro de 2022, ocorreu a formação dos grupos e a aplicação do Programa de oito semanas de *Hatha-Yoga*. Assim, dos 300 alunos elegíveis para essa etapa, foi originada uma lista aleatória e numerada.

Devido ao risco de desistências, para essa fase optou-se pela realização de um convite, via *e-mail*, enviado pelos coletores, a cada um dos 300 alunos que atenderam aos critérios de elegibilidade, a fim de averiguar a disponibilidade em participar da intervenção, de acordo com os horários que a *Hatha-Yoga* seria ofertada, sendo estabelecido no máximo três tentativas de contato. A lista seguiu a lógica de, caso não aceite, o próximo era convidado. Houve a recusa de 264 estudantes em participar da etapa da intervenção, pela incompatibilidade de horário, e a partir do baixo quantitativo de aceites, não houve randomização aleatória. Nessa fase, denominada de Tempo Zero, 36 discentes aceitaram participar da intervenção, e os grupos foram preenchidos por alocação simples, de 1:1, organizado por um membro da pesquisa (coletador), a partir da sequência de aceitação, constituindo dois grupos, Grupo Intervenção (GI) (n=18) e Grupo Controle (GC) (n=18), ou seja, o primeiro aceite foi alocado no GI, o segundo no GC e assim sucessivamente, até a finalização da lista de participantes.

Optou-se, neste estudo, por um GC inativo (que recebeu a intervenção após o término do estudo), pois condições inativas possibilitam a detecção dos resultados da intervenção real, ademais, pela dificuldade de se identificar uma intervenção placebo adequada que potencialmente não exercesse efeito nos indicadores emocionais avaliados^(67)^.

Ainda, durante as primeiras quatro semanas do Programa, também denominado de Tempo Um, houve quatro desistências do GI, resultando, ao final do estudo, na seguinte alocação: GI (n = 14) e GC (n = 18) (Figura 1).

**Figura 1 -:** Fluxograma de recrutamento e alocação dos participantes baseado nas orientações do CONSORT (2010). Cuiabá, MT, Brasil, 2022

### Intervenção

A intervenção deste estudo consistiu em um programa de oito semanas de *Hatha-Yoga*. As sessões foram ministradas pela primeira autora, que é instrutora certificada em *Hatha-Yoga* com aperfeiçoamentos na abordagem de *Yoga* experiencial e que não participou das etapas de recrutamento e avaliação dos participantes. O programa executado foi previsto para praticantes leigos e iniciantes em *yoga*, consistindo em 16 sessões de 60 minutos de aula prática e 15 minutos de aula teórica, totalizando 80 minutos, duas vezes por semana, no período de junho a outubro de 2022, em um espaço no *campus* da instituição participante.

O Programa de *Hatha-Yoga* foi sistematizado por meio de um protocolo, conforme diretrizes prévias^(68)^, combinando técnicas inspiradas no *Hatha-Yoga* antigo, pré-moderno e contemporâneo, como os sons sagrados (*mantras*), espreguiçamento, alongamento, relaxamento, posturas físicas (*asanas*), práticas de limpeza (*kriyas*), dobras (*mudras*), contrações direcionadas (*bandhas*), práticas respiratórias (*pranayama*), intenção compassiva (*sankalpa*), meditação e relaxamento, com duração total de 60 minutos^(45-69-70)^.

Durante o desenvolvimento do programa, os estudantes foram incentivados a estarem presentes nas sessões para a continuidade na pesquisa. O GI foi subdividido em quatro horários ao longo da semana, com o intuito de reter os participantes, além da possibilidade de reposição, em caso de ausência, sempre que necessário. Os participantes do GC não receberam a intervenção, contudo, após o término da pesquisa, por razões éticas, foram convidados para um novo ciclo do programa de oito semanas de *Hatha-Yoga*.

### Coleta de dados

Os dados foram coletados de maneira majoritariamente presencial e em dois momentos distintos: fase pré-intervenção, realizada entre maio e junho de 2022, e fase pós-intervenção, realizada em outubro de 2022. Na primeira fase, dez coletores previamente treinados compareceram às salas de aula e, após autorização do professor presente, apresentaram detalhes do estudo aos estudantes e os convidaram a participar da pesquisa/intervenção. Aos estudantes que aceitaram responder aos questionários, foi apresentado o Termo de Consentimento Livre e Esclarecido (TCLE) e foi orientado a assiná-lo, após leitura atenta, indicando ciência e interesse em participar da investigação. O preenchimento dos questionários, nessa fase, durou cerca de 45 minutos.

Na fase pós-intervenção, após as oito semanas de práticas de *Hatha-Yoga* com o GI, os mesmos instrumentos foram novamente aplicados aos concluintes do GI ao término da prática final, sob supervisão dos coletores previamente treinados, que os acondicionaram em envelopes lacrados sem identificação de grupos. Concomitantemente, os participantes do GC foram contactados via *e-mail*, pela equipe coletora, e receberam um link do *Google Drive* com a versão dos formulários. Apenas essa etapa foi realizada de maneira remota.

Os dados brutos passaram por dupla digitação e comparação por meio do *website Diffchecker* ^®^, sendo armazenados nos *softwares Word* ^®^, *Excel* ^®^ e *Google Drive*.

### Análise dos dados

Os dados foram tratados de maneira descritiva e inferencial. Nas análises descritivas, variáveis categóricas foram apresentadas por meio de suas frequências absoluta e relativa, enquanto variáveis contínuas foram apresentadas por meio de seus escores médios e respectivos desvios-padrão. Nas análises inferenciais, comparações entre proporções foram realizadas por meio do teste de Qui-Quadrado ou Exato de Fisher com extensão de Freeman-Halton, quando necessário. Para comparações de médias, foram utilizados testes t para amostras pareadas nas análises intragrupos e testes t para amostras independentes nas análises entre grupos. Os tamanhos de efeito foram calculados por meio do teste g de Hedges. Os intervalos de confiança de 95% (IC95%) foram obtidos por meio de procedimentos de *bootstrapping* (1000 reamostragens), que fornecem IC95% corrigido e acelerado por viés (*bias-corrected and accelerated* - BCa IC95%). A técnica foi utilizada a fim de corrigir os desvios da normalidade da distribuição amostral e minimizar as diferenças entre os tamanhos dos grupos^(71)^. As análises foram realizadas por meio do *softwareStatistical Package for the Social Sciences* (SPSS), versão 27.

### Aspectos éticos

Este estudo foi aprovado pelo Comitê de Ética em Pesquisa com seres humanos sob o parecer nº 49695621.9.0000.8124 e respeitou as diretrizes referentes a pesquisas em humanos da Resolução 466/2012 do Conselho Nacional de Saúde e os princípios da Declaração de Helsinki. O estudo foi inserido em dezembro de 2021 no Registro Brasileiro de Ensaios Clínicos (ReBEC) sob o código de registro RBR-10jgv7ky.

## Resultados

Iniciaram a intervenção proposta neste estudo 36 participantes, que estavam alocados igualmente entre GI (n = 18) e GC (n = 18) e que se demonstraram homogêneos (p > 0,05) em relação às características sociodemográficas, acadêmicas e à maioria dos indicadores emocionais avaliados neste estudo (Tabela 1), com exceção dos níveis de autocompaixão, os quais mostraram-se mais elevados nos estudantes do GC (p = 0,005).

O efeito da intervenção nos IE dos participantes do GI (comparação de escores médios pré e pós-intervenção) foi apresentado na Tabela 2.

**Tabela 1 -:** Caracterização sociodemográfica, acadêmica e indicadores emocionais de estudantes de graduação da área de saúde segundo grupos de alocação no período pré-intervenção. Cuiabá, MT, Brasil, 2022

**Variáveis**	**Grupo Intervenção (n** * **= 18)**	Grupo Controle (n* = 18)	**Valor de p**
	n* (%)	n* (%)	
Sexo			
Feminino	13 (72,2%)	17 (94,4%)	0,177 ^†^
Masculino	05 (27,8%)	01 (5,6%)	
Idade ^‡^	22,06 (±2,31)	23,72 (±7,74)	0,388 (-5,268; 1,449) ^§^
Cor da pele autodeclarada			
Não branca	16 (88,9%)	12 (66,7%)	0,228 ^†^
Branca	02 (11,1%)	06 (33,3%)	
Orientação sexual			
Heterossexual	14 (77,8%)	15 (83,3%)	1,000 ^†^
Orientações sexuais minoritárias	04 (22,2%)	03 (16,7%)	
Prática religiosa			
Sim	08 (44,4%)	12 (66,7%)	0,180
Não	10 (55,6%)	06 (33,3%)	
Status conjugal			
Com companheiro	03 (16,7%)	03 (16,7%)	1,000 ^†^
Sem companheiro	15 (83,3%)	15 (83,3%)	
Status laboral			
Trabalha e estuda	05 (27,8%)	02 (11,1%)	0,402 ^†^
Somente estuda	13 (72,2%)	16 (88,9%)	
Status de moradia			
Mora sozinho(a)	03 (16,7%)	01 (5,6%)	0,603 ^†^
Não mora sozinho(a)	15 (83,3%)	17 (94,4%)	
Curso			
Enfermagem	06 (33,3%)	10 (55,6%)	0,342 ^||^
Medicina	01 (5,6%)	02 (11,1%)	
Nutrição	04 (22,2%)	01 (5,6%)	
Psicologia	07 (38,9%)	05 (27,8%)	
Atenção plena ^‡^	49,21 (±12,84)	54,17 (±12,07)	0,272 (-14,208; 4,030) ^§^
Compaixão ^‡^	25,57 (±7,42)	28,11 (±5,84)	0,287 (-6,343; 2,181) ^§^
Autocompaixão ^‡^	68,29 (±12,46)	79,83 (±9,16)	0,005 (-17,877; -3,317) ^§^
Depressão ^‡^	15,71 (±6,00)	13,72 (±4,62)	0,297 (-2,574; 4,883) ^§^
Ansiedade ^‡^	14,71 (±4,48)	13,44 (±4,69)	0,445 (-2,062; 3,520) ^§^
Estresse ^‡^	17,36 (±3,71)	16,83 (±4,73)	0,736 (-2,730; 3,005) ^§^
Risco de suicídio ^‡^	0,64 (±1,15)	0,44 (±1,29)	0,655 (-0,387; 1,333) ^§^
Impulsividade ^‡^	68,07 (±15,27)	65,89 (±10,90)	0,640 (-6,017; 9,799) ^§^
Sonolência excessiva ^‡^	10,57 (±4,07)	9,44 (±3,85)	0,430 (-2,484; 3,417) ^§^

^*^n = Tamanho amostral; ^†^Valor de p obtido por meio do teste Exato de Fisher; ^‡^Para caracterização de variáveis contínuas apresentou-se médias e desvios-padrão; ^§^Valor de p obtido por meio de teste t para amostras independentes, com IC95% obtido por meio de técnica de *bootstrapping* com 1000 reamostragens; ^||^Valor de p obtido por meio do teste Exato de Fisher com extensão de Freeman-Halton para tabelas maiores que 2x2

**Tabela 2 -:** Comparação de escores médios de indicadores emocionais de estudantes alocados no grupo intervenção nos momentos pré e pós-intervenção (n = 14). Cuiabá, Mato Grosso, Brasil, 2022

**Indicadores emocionais**	**Grupo Intervenção**					
	**Pré-Intervenção (n=14)**	**Pós-Intervenção (n=14)**	**t**	**Valor de p**	BCa IC95%*	**Tamanho de efeito (g de Hedges)**
	**Média (dp** ^†^ **)**	**Média (dp** ^†^ **)**				
Atenção plena disposiocional	49,21 (±12,84)	52,29 (±14,96)	1,072	0,288	-8,783; 4,699	0,21
Compaixão	25,57 (±7,42)	27,93 (±5,27)	2,168	**0,050**	-4,714; -0,057	0,35
Autocompaixão	68,29 (±12,46)	81,14 (±18,86)	3,165	**0,014**	-20,643; -4,084	0,77
Sintomas depressivos	15,71 (±6,00)	13,07 (±5,38)	1,459	0,171	-1,286; 6,357	0,43
Sintomas ansiosos	14,71 (±4,48)	12,50 (±4,29)	2,302	**0,032**	0,143; 4,348	0,47
Estresse	17,36 (±3,71)	16,57 (±2,34)	0,738	0,507	-1,148; 2,863	0,24
Risco de suicídio atual	0,64 (±1,15)	1,64 (±5,11)	0,717	0,550	-3,929; 0,643	0,30
Impulsividade	68,07 (±15,27)	66,43 (±16,48)	0,733	0,500	-1,929; 5,237	0,10
Sonolência diurna	10,57 (±4,07)	9,21 (±3,91)	2,464	**0,045**	0,357; 2,400	0,32

*BCa IC95% = Intervalo de confiança de 95% corrigido e acelerado por viés (*bias-corrected and accelerated*); **†**dp = Desvio-padrão

Evidencia-se aumento nos níveis de compaixão (p<0,050) e de autocompaixão (p<0,014), bem como a redução dos sintomas ansiosos (p<0,032) e da sonolência diurna (p< 0,045) após a conclusão do programa de oito semanas de *Hatha-Yoga*.

No mesmo período, os participantes alocados no grupo que não recebeu a intervenção (GC) apresentaram decréscimo do indicador emocional de atenção plena disposicional (p<0,013), sem evidências de melhoras ou pioras nos demais IE avaliados (Tabela 3).

**Tabela 3 -:** Comparação de escores médios de indicadores emocionais de estudantes alocados no grupo controle nos momentos pré e pós-teste (n = 18). Cuiabá, MT, Brasil, 2022

**Indicadores emocionais**	**Grupo Controle**					
	**Pré-teste (n=18)**	**Pós-teste (n=18)**	**t**	**Valor de p**	BCa IC95%*	**Tamanho de efeito (g de Hedges)**
	**Média (dp** ^†^ **)**	**Média (dp** ^†^ **)**				
Atenção plena disposicional	54,17 (±12,07)	43,72 (±12,87)	3,235	**0,013**	3,778; 16,919	0,80
Compaixão	28,11 (±5,84)	27,00 (±8,36)	0,814	0,427	-1,426; 3,722	0,15
Autocompaixão	79,83 (±9,16)	73,61 (±13,83)	0,218	0,134	-6,121; 4,255	0,52
Sintomas depressivos	13,72 (±4,62)	14,56 (±5,78)	0,753	0,440	-7,167; 0,056	0,15
Sintomas ansiosos	13,44 (±4,69)	11,94 (±4,56)	1,205	0,281	-2,778; 1,116	0,31
Estresse	16,83 (±4,73)	14,33 (±5,16)	1,676	0,138	-0,444; 3,556	0,48
Risco de suicídio atual	0,44 (±1,29)	3,33(±8,64)	1,401	0,279	-0,222; 5,526	0,55
Impulsividade	65,89 (±10,90)	68,11 (±11,41)	1,481	0,193	-5,608; 0,667	0,19
Sonolência diurna	9,44 (±3,85)	10,94 (±4,54)	1,448	0,165	-3,489; 0,500	0,34

*BCa IC95% = Intervalo de confiança de 95% corrigido e acelerado por viés (*bias-corrected and accelerated*); **†**dp = Desvio-padrão

Por fim, a Tabela 4 compara os escores médios de IE de estudantes alocados no GI e GC pós-teste e nela não se observaram diferenças estatisticamente significativas nestes parâmetros, entretanto, destacam-se as diferenças entre escores médios dos níveis autocompaixão e atenção plena disposicional que apresentaram os maiores tamanhos de efeitos, respectivamente g = 0,66 e g = 0,60, condizentes com tamanhos de efeitos moderados.

**Tabela 4 -:** Comparação de escores médios de indicadores emocionais de estudantes alocados no grupo intervenção (n = 14) e no grupo controle (n = 18) no momento pós-teste. Cuiabá, MT, Brasil, 2022

**Indicadores emocionais**	**Pós-teste**					
	**Grupo Intervenção (n = 14)**	**Grupo Controle (n = 18)**	**t**	**Valor de p**	BCa IC95%*	**Tamanho de efeito (g de Hedges)**
	**Média (dp** ^†^ **)**	**Média (dp** ^†^ **)**				
Atenção plena disposicional	52,29(±14,96)	43,72 (±12,87)	1,739	0,089	-1,280; 19,238	0,60
Compaixão	27,93(±5,27)	27,00 (±8,36)	0,363	0,702	-3,850; 6,249	0,13
Autocompaixão	81,14(±18,86)	73,61 (±13,83)	1,575	0,104	-2,297; 18,024	0,66
Sintomas depressivos	13,07(±5,38)	14,56 (±5,78)	0,742	0,522	-5,287; 2,696	0,26
Sintomas ansiosos	12,50(±4,29)	11,94 (±4,56)	0,351	0,464	-2,555; 3,999	0,12
Estresse	16,57 (±2,34)	14,33 (±5,16)	1,504	0,728	-0,581; 5,129	0,52
Risco de suicídio atual	1,64 (±5,11)	3,33(±8,64)	0,648	0,143	-6,413; 2,631	0,22
Impulsividade	66,43 (±16,48)	68,11 (±11,41)	0,341	0,735	-9,901; 6,092	0,12
Sonolência diurna	9,21(±3,91)	10,94 (±4,54)	1,135	0,266	-4,648; 1,167	0,39

*BCa IC95% = Intervalo de confiança de 95% corrigido e acelerado por viés (*bias-corrected and accelerated*); **†**dp = Desvio-padrão

## Discussão

Este estudo-piloto analisou se indicadores emocionais de estudantes universitários poderiam ser alterados após a realização de um programa de oito semanas de *Hatha-Yoga*. Apesar da ausência de diferenças estatisticamente significativas entre o GI e o GC no período pós-teste, evidenciou-se, entre os estudantes que participaram da intervenção, melhoras em alguns IE. Achados congruentes com pesquisas prévias apontavam a prática yóguica como potencialmente benéfica para a regulação emocional^(14)^, o equilíbrio de reatividades emocionais e sentimentos/afetos depreciativos^(53-63-72-73)^, pois práticas corpo-mente que cultivam a compaixão ou bondade amorosa contribuem para a manutenção, expansão e a melhoria das emoções positivas e o fortalecimento de comportamentos interpessoais/pró-sociais favoráveis^(53-64-74-75)^.

Neste estudo, a prática de *Hatha-Yoga* seguiu um protocolo de intervenção que integrou técnicas psicofísicas yóguicas e um momento de acolhimento dos indivíduos para trocas teórico-educativas sobre a ética social do *yoga* (*yamas*) e as observâncias de si (*niyamas*), bem como o compartilhamento de experiências sobre o cuidado de si ampliado, portanto, coletivo e afetivo^(23-24-76-77)^. A prática foi aplicada duas vezes por semana, durante oito semanas, contribuindo para o aumento das capacidades de compaixão e autocompaixão dos estudantes da amostra, ao mesmo tempo em que diminuiu a severidade de sintomas ansiosos e sonolência diurna neste grupo.

A compaixão, de modo geral, pode estimular a calma, contato afetivo e afiliação^(49-78-79)^ e, a longo prazo, pode vincular propósitos de vida, sentimentos de proximidade, afetos positivos e comportamentos de conexão coletiva^(75)^. Assim, a compaixão pode ser modulada, por meio de intervenções que a estimulam e produzem benefícios intrapessoais físicos e mentais, interpessoais, além de cooperação e tolerância para com o sofrimento alheio^(80-82)^, aspectos fundamentais para profissionais em formação na área da saúde^(38)^. Por conseguinte, as práticas que proporcionam o cultivo da compaixão podem ser um meio e um aliado para atenuar o sofrimento, promover um florescimento individual e social^(48-83-86)^.

A autocompaixão é a capacidade de conscientemente ter compaixão por si, e de amabilidade em simbiose com a humanidade compartilhada e a atenção plena^(87-88)^. A autocompaixão não só desperta a habilidade de enfrentamento intrapessoal, como também pode sensibilizar para a maior aceitação e busca por apoio social nos momentos de desafios e de sofrimento emocional^(81-89)^.

O aumento nos níveis de autocompaixão encontrado no GI deste estudo é concernente aos resultados encontrados na literatura sobre práticas corpo-mente que demonstram a sua contribuição positiva para a modulação da autocompaixão^(87-90)^. Além disso, resultados semelhantes foram observados em um estudo experimental randomizado com 100 estudantes de enfermagem indianos, utilizando o mesmo tipo e período de intervenção. Nele, verificou-se que o *yoga* foi eficaz na melhora da autocompaixão e da atenção plena na comparação entre grupos (intervenção e controle)^(60)^. Outra investigação quasi-experimental com estudantes de graduação em enfermagem norte-americanos (n=73), objetivando promover a autocompaixão, ofertou aulas semanais de *yoga* (uma hora) durante 12 semanas e constatou que o GI apresentou maior cultivo da amabilidade/autobondade, uma das facetas primordiais da autocompaixão, do que o GC^(40)^. Neste estudo não se encontraram diferenças estatisticamente significativas entre os níveis de autocompaixão entre GI e GC no pós-teste, entretanto, o tamanho de efeito da diferença das médias foi moderado, o que, coerentemente com os achados acima, pode indicar um efeito importante da prática de *Hatha-Yoga* no IE, que não foi evidenciado devido ao pequeno poder amostral deste estudo.

O aumento dos níveis de compaixão e autocompaixão fortalece o processo formativo dos estudantes universitários para além da aquisição de competências clínicas e gerenciais, à medida que potencializa a corresponsabilidade e a autonomia para o cuidado de si e, principalmente, motiva a formação de redes de apoio, como amigos, colegas, professores, familiares, grupos de apoio, entre outras^(13-91-92)^. A autocompaixão possibilita um cuidar-se ampliado, uma vez que agencia redes sociomateriais diversas, fortalecendo vínculos fundamentais para profissionais diretamente envolvidos no cuidado humano. Cuidar do outro implica cuidar de si, e as redes de apoio são elementos fundamentais nesse processo^(76-77-92)^.

Indicadores Emocionais que diminuíram no GI após a prática de *Hatha- Yoga*, os sintomas ansiosos são fenômenos de ordem mental e subjetiva ou provenientes de experiências corpóreas estimuladas e aprendidas distorcidamente no caótico cotidiano contemporâneo. Os sintomas acionam o sistema de alerta no cérebro, via eixo hipotálamo-pituitária-adrenal (HPA)^(49-93-94)^, o qual ativa o mecanismo de “luta e fuga”. Esse mecanismo, que antes, em estado de homeostase, era esporadicamente acionado, com a ativação do HPA, passa a ficar em um estado de permanente ativação, sobrecarregando o indivíduo de reatividade e subjetividades depreciativas, desatenção, irritabilidade e impulsividade que, na maioria das vezes, são desproporcionais à realidade^(93)^. Intervenções com *Hatha-Yoga* aplicadas em estudantes universitários reafirmam o potencial da prática em mitigar a severidade de sintomatologia ansiosa^(95)^.

Em relação à sonolência diurna, embora esta não seja uma emoção no sentido exato, a sua presença pode resultar negativamente na qualidade de vida e se constituir em um indicativo de irregularidade cognitiva, comportamental, mental e emocional para estudantes universitários^(RYFF, 2023-96-97)^. A sonolência diurna pode ser conceituada como a capacidade reduzida de um indivíduo em permanecer acordado e em alerta durante as horas normais do dia, resultando em cochilos e lapsos de sonolência ou sono^(98-99)^.

Para a compreensão yóguica, os sofrimentos (*duhkha-traya*) do sono são incongruências psicofísicas (*viksepa*) provenientes de percepções erradas (*bhrantidarsana*) dos estímulos internos e externos (*klesas*) pelo indivíduo e da sua dificuldade em manter a homeostasia entre corpo e mente (*manas*)^(100)^. Independente do olhar para o fenômeno, pesquisas apontam que a sonolência diurna pode prejudicar significativamente a saúde mental de universitários no processo de formação acadêmica^(101-102)^.

Em um estudo de revisão sistemática e meta-análise, referente à qualidade de sono e insônia, foi apontado que práticas como *yoga* podem reduzir a sonolência diurna em adultos^(103)^. Outra revisão sistemática sobre aplicações clínicas de estudos experimentais com práticas meditativas baseadas em *mindfulness, yoga* e meditação transcendental, apontou desfecho positivo na melhora da qualidade de sono em jovens e adultos^(104)^. As intervenções corpóreo-mentais contribuem para a sincronização neuronal e, consequentemente, para a melhora das funções globais básicas de praticantes^(87-49)^. Dessa maneira, a meditação yóguica pode desempenhar um papel de regulação não apenas no desempenho cognitivo, como também na promoção de uma restauração corpóreo-sensório-mental integral^(104)^.

Um achado importante do estudo se refere ao indicador de atenção plena disposicional (APD), que embora tenha se mantido estável no GI na comparação pré e pós-teste, diminuiu significativamente no GC no mesmo período. Além disso, destaca-se que apesar da ausência de significância estatística na análise entre grupos, intervenção e controle, no pós-teste o tamanho de efeito encontrado foi moderado, denotando uma diferença importante entre os valores dos escores médios analisados, o que pode ser clinicamente relevante. Uma possibilidade para esse fato é que o grupo intervenção pode ter se beneficiado da prática de *Hatha-Yoga* e, mesmo não contribuindo para o aumento dos níveis de APD, atuou evitando sua diminuição como evidenciado no GC.

O processo atencional compreende um ciclo no qual o indivíduo constantemente alterna entre atenção, divagação, consciência da divagação e retorno ao foco^(105-106)^. Nesse processo, a divagação pode gradualmente ser amenizada à medida que se cultivam experiências atencionais por meio de práticas meditativas^(49-82-107-108)^.

Embora ainda sejam reduzidas as publicações científicas sobre intervenção com *yoga* para avaliação comparativa da APD em estudantes universitários, evidências disponíveis sugerem melhorias nos níveis de APD após a implementação de intervenções yóguicas, provavelmente devido à melhora da concentração e à redução da divagação mental proveniente de práticas diárias^(90-109-110)^.

Importante destacar que a APD está presente no cultivo de compaixão e a autocompaixão, e os estudos têm cada vez mais apontado correlações positivas destas com a capacidade atencional disposicional^(29-79-108)^, bem como podem contribuir para a regulação emocional de sintomas ansiosos^(111)^ e modular a qualidade do sono^(41)^.

Este estudo não é isento de limitações. Destaca-se o fato de ter-se empregado a alocação simples dos participantes por ordem de aceitação na fase de constituição dos grupos controle e experimental, pois tal fato pode ter atuado na dificuldade de mensurar os reais efeitos da intervenção nos indicadores emocionais avaliados. Além disso, aponta-se, ainda, a falta de seguimento (atrito) dos participantes durante a fase de aplicação do protocolo de oito semanas de *Hatha-Yoga*, mesmo tendo-se adotado estratégias de retenção como a flexibilização de horários para realização das práticas e o envio de lembretes semanais e mensagens de estímulo aos participantes do grupo intervenção. O atrito é um desafio ímpar em avaliações longitudinais pode comprometer significativamente a validade e integridade de uma investigação. Sugerem-se novos estudos randomizados, com maior tamanho amostral que compensem as perdas de seguimento e que acompanhem os estudantes ao longo do processo formativo e incluam outros estudantes além daqueles da área da saúde.

Outra limitação importante refere-se ao tamanho amostral recrutado, que ficou aquém do mínimo estipulado no cálculo amostral, o que pode ter contribuído para que possíveis efeitos da intervenção sobre os indicadores emocionais não tenham sido detectados. Assim, para mitigar essa limitação, os autores optaram por apresentar e discutir os resultados por meio de medidas de tamanho de efeito que demonstram quão fortes são os achados do estudo. Por fim, a adoção de um grupo controle inativo pode ser vista como uma limitação, sendo sugerido que estudos futuros possam incluir um grupo controle ativo de modo a fornecer comparações dos efeitos da prática de *Hatha-Yoga* com outras abordagens integrativas e complementares.

Como contribuição, o estudo avança no conhecimento científico, ao propor uma intervenção não farmacológica como forma de cuidado à saúde mental de estudantes, no contexto universitário, com resultados significativos na modulação dos IE que, quando equilibrados, repercutem em melhores desempenhos acadêmicos e em melhores respostas adaptativas às demandas do cotidiano universitário. Ademais, o *Hatha-Yoga* é uma prática integrativa que fortalece indivíduos no âmbito pessoal e interpessoal/pró-social, tornando-se mais segura e responsável, quando baseada em evidências científicas.

Por fim, ressalta-se que os resultados devem ser interpretados com cautela, pois, sendo um estudo-piloto, eles fornecem subsídios para o planejamento de pesquisas futuras, em vez de constituírem uma conclusão definitiva.

## Conclusão

Este estudo teve como objetivo avaliar preliminarmente os IE de estudantes de graduação da área da saúde pré e pós-intervenção com um programa de oito semanas de *Hatha-Yoga,* resultando em aumento significativo da capacidade de compaixão, autocompaixão, bem como na redução de sintomas ansiosos e sonolência diurna no grupo intervenção, após oito semanas de aplicação de um programa de *Hatha-Yoga*. No grupo controle houve um decréscimo significativo dos níveis de APD. Apesar de não terem sido encontradas diferenças estatisticamente significativas nos IE do GI e GC no pós-teste, evidenciou-se que as diferenças entre as médias de autocompaixão e APD entre esses dois grupos tiveram tamanho de efeito moderado, o que pode sugerir diferenças clinicamente relevantes.

Nessa perspectiva, instituições de ensino superior devem considerar o desenvolvimento de intervenções não farmacológicas, restaurativas, como as práticas integrativas e complementares em saúde, sobretudo as do tipo corpóreo-sensório-mental como o *Hatha-Yoga,* que incorporam a motivação do reconhecimento e cultivo integrado da atenção plena, capacidade de compaixão e autocompaixão, para apoiar o cuidado de si ampliado dos estudantes universitários. Estudos futuros que incluam uma população amostral maior e acompanhamento a longo prazo são necessários para assegurar um efeito benéfico e suas repercussões.

# Translation: Indicadores emocionales de estudiantes universitarios: un estudio piloto cuasiexperimental con *Hatha-Yoga* *

**DOI:** 10.1590/1518-8345.7193.4589

**Authors:** Vanessa Ferraz Leite, Moisés Kogien, Tatiane Lebre Dias, Margani Cadore Weis Maia, Larissa de Almeida Rézio, Samira Reschetti Marcon

**Affiliations:** 1 Universidade Federal de Mato Grosso, Faculdade de Enfermagem, Cuiabá, MT, Brasil.; 2 Becaria de la Coordenação de Aperfeiçoamento de Pessoal de Nível Superior (CAPES), Brasil.; 3 Universidade Federal de Mato Grosso, Departamento de Educação, Cuiabá, MT, Brasil.

**Keywords:** Yoga, Emociones, Estudiantes, Universidades, Salud Mental, Estudios Cuasi Experimentales

## Abstract

analizar los efectos de un programa de *Hatha-Yoga* en los indicadores emocionales de estudiantes de grado.

estudio piloto cuasiexperimental, desarrollado con 36 estudiantes, con una muestra final compuesta por 14 en el grupo de intervención y 18 en el grupo de control, totalizando 32. Los indicadores emocionales de los participantes fueron evaluados antes y después de la intervención de *Hatha-Yoga* mediante la *Mindful Attention Awareness Scale*, *Santa Clara Brief Compassion Scale*, *Self-Compassion Scale*, *Mini International Neuropsychiatric Interview*, *Depression*, *Anxiety*, *and Stress Scale*, *Barratt Impulsiveness Scale* y *Epworth Sleepiness Scale*. Se realizaron análisis descriptivos e inferenciales con pruebas t (pareadas y para grupos independientes) para evaluar el efecto de la intervención.

en los análisis intragrupos, se observó en el grupo de intervención un aumento en los indicadores emocionales de compasión y autocompasión, así como una disminución de la ansiedad y la somnolencia diurna tras ocho semanas de práctica de *Hatha-Yoga*. En el grupo de control, se registró un descenso significativo de la atención plena disposicional. En el análisis entre grupos en la fase postest se encontraron diferencias con tamaños de efecto moderados para autocompasión y atención plena disposicional.

la práctica de *Hatha-Yoga* impactó positivamente en los indicadores emocionales de los estudiantes, contribuyendo a la promoción de la salud y al cuidado de sí ampliado (RBR-10jgv7ky).

## Destacados

(1) Impacto positivo en los indicadores emocionales de los participantes. (2) Disminución de los efectos de los indicadores negativos de los participantes. (3) Contribución a la promoción de la salud y al cuidado de sí ampliado. (4) Reducción de la atención plena disposicional en ausencia de la práctica yóguica.

## Introducción

Las emociones son reacciones internas, involuntarias y observables, que implican manifestaciones físicas, como alteraciones cardiorrespiratorias, secreciones hormonales, expresiones faciales y cambios posturales, desencadenadas por eventos sensoriomotores o rememorados, cuyo papel primordial es mantener la vida, identificar amenazas o indicar logros individuales o sociales^(1)^. Aunque innatas, las emociones pueden ser moldeadas según el contexto social y los aspectos culturales. Estados emocionales como alegría, tristeza, miedo, ira, sorpresa o repugnancia son considerados emociones universales^(2-3)^.

Con la progresiva complejización de las experiencias emocionales hacia sentimientos, y la comprensión de estos como eventos mentales conscientes que representan simultáneamente experiencias del cuerpo^(4-5)^, así como las diferentes condiciones que influyen en su desencadenamiento y percepción regulatoria, el contexto universitario surge como un importante impulsor de las emociones y los sentimientos, al tiempo que favorece el desarrollo de habilidades sociales y la adquisición de competencias profesionales y personales, mediante nuevos ritmos de estudio, normas y reglas colectivas^(6-7)^. Esta combinación de emociones y sentimientos puede potenciar el desarrollo de habilidades personales y prosociales, pero también puede generar sufrimiento y señalar respuestas desadaptativas con un progresivo agravamiento emocional desproporcionado en determinadas situaciones^(8-11)^.

Desde esta perspectiva, estudios señalan que la capacidad de atención plena, así como las habilidades para reconocer y expresar compasión por uno mismo y por los demás, están directa e indirectamente asociadas con la regulación emocional y una mejor respuesta ante el sufrimiento mental^(2,12-15)^. Además, la regulación emocional puede ser un mecanismo de cambio en la relación entre capacidades atencionales, compasivas y salud mental^(16-18)^.

La Organización Mundial de la Salud (OMS) estima un aumento de más del 25% en los síntomas comunes de depresión y ansiedad en la población mundial desde 2020, lo que indica una crisis de salud mental global y resalta la importancia de intervenciones adecuadas ante situaciones altamente estresantes que requieren respuestas adaptativas rápidas^(19)^.

Los estudiantes universitarios experimentan altos niveles de sufrimiento mental, exacerbados por la desafiante transición de la educación secundaria a la educación superior, las presiones académicas y la adaptación social y cultural^(20-22)^. Entre ellos, los estudiantes del área de la salud sufren un impacto emocional aún mayor^(23)^ debido a la sobrecarga de demandas personales y académicas, así como a su rol en el cuidado de los demás, lo que exige un alto rendimiento, promoviendo la autocompetencia y la competitividad^(23-25)^. En consecuencia, la prevalencia combinada de síntomas de depresión y ansiedad entre estudiantes universitarios alcanza el 33,6%^(26)^, lo que resulta alarmante y refleja la urgencia de prestar una atención cuidadosa a la salud mental de esta población. Además, alrededor del 14% de los universitarios presentan desenlaces relacionados con el comportamiento suicida^(27)^ y padecen problemas en la calidad del sueño, insomnio y estrés, fenómenos interconectados que afectan su rendimiento académico y su salud mental^(28)^.

En la búsqueda de comprender las emociones y sentimientos experimentados por los jóvenes universitarios, denominados indicadores emocionales (IE), se destacan variables que permiten evaluar las reacciones subjetivas y objetivas asociadas con los aspectos emocionales basales, primarios o universales (afecciones activadas por las vías somatosensoriales vinculadas a los nervios aferentes), así como los sentimientos emocionales, como la atención plena, la compasión y la autocompasión (afectos establecidos por las vías perceptivas conectadas a los nervios eferentes)^(2,29-30)^.

Los IE evidencian condiciones que pueden repercutir en problemas graves de salud mental, como depresión, ansiedad, estrés crónico y alteraciones del sueño, frecuentemente presentes en estudiantes del área de la salud^(10,31)^. No obstante, también pueden desempeñar un papel beneficioso al favorecer el autocuidado, el autoconocimiento y la prosociabilidad^(32-34)^.

Estos IE pueden regularse mediante la implementación de estrategias, como intervenciones farmacológicas y no farmacológicas^(35)^. Las intervenciones no farmacológicas, como el ejercicio físico y las prácticas cuerpo-mente, han demostrado eficacia en la mejora de la salud mental de los estudiantes universitarios, fortaleciendo IE prosociales y de autocuidado, como la atención plena, la compasión y la autocompasión^(36-40)^, además de proporcionar mejores respuestas adaptativas cuerpo-mente a los síntomas de depresión, ansiedad y estrés, la impulsividad y la somnolencia diurna^(41-43)^.

El *Hatha-Yoga* es una de las formas antiguas de yoga que reintegra el cuerpo, la mente y la intuición espiritual. Sus principales objetivos son el fortalecimiento corporal y la estabilización mental, con el fin de propiciar experiencias de profunda transformación sensitiva e intuitiva^(21,23)^. En la vida contemporánea, el *Hatha-Yoga* destaca como una herramienta potencial para abordar el bienestar emocional y psicológico de los estudiantes, con la capacidad de promover un cuidado restaurativo que integra cuerpo y mente^(44-46)^ y que, en muchos casos, se ve fortalecido por prácticas que enfatizan la resiliencia, la atención plena, la compasión y la autocompasión^(47-49)^.

Los programas de *Hatha-Yoga* que fomentan experiencias compasivas pueden contribuir a la modulación de los IE^(31,50-53)^ y pueden practicarse de manera individual y/o colectiva, en entornos interiores o exteriores, proporcionando una experiencia de reparación emocional a través de una desprogramación y reprogramación autonómica del cuerpo, inseparable de la mente, además de una revitalización global de la vida. Esta revitalización ocurre a partir de la creación de nuevas realidades ritualísticas, con el descanso y la restauración corporal, sensorial, gestual, mental y espiritual^(54-59)^.

En este sentido, las investigaciones señalan beneficios psicosomáticos de las prácticas yóguicas para estudiantes universitarios^(38,60)^. Sin embargo, existen pocos estudios de intervención con un nivel de evidencia moderado a alto que evalúen simultáneamente diversos IE en esta población^(41)^.

Ante lo expuesto, como un estudio piloto, se pretende analizar los efectos de un programa de *Hatha- Yoga* en los indicadores emocionales de estudiantes de grado, con el fin de proporcionar información para la planificación de futuras investigaciones confirmatorias con mayor control y tamaño muestral. Se parte de la hipótesis de que la exposición a un programa de *Hatha-Yoga* podría regular indicadores emocionales como la atención plena disposicional, la compasión y la autocompasión, además de reducir síntomas depresivos, ansiosos, de estrés, impulsividad, somnolencia diurna y el riesgo de suicidio entre los estudiantes participantes en la intervención.

## Método

### Diseño del estudio

Se trata de un estudio piloto cuasiexperimental, del tipo antes y después, con asignación de grupo de control y enmascaramiento simple (estadístico), basado en las recomendaciones del *Consolidated Standards of Reporting Trials* (CONSORT)^(61)^ para estudios piloto.

### Escenario

Este estudio piloto se llevó a cabo en un espacio cubierto, amplio y ventilado de una universidad pública federal ubicada en una capital de la región centro-oeste de Brasil. Dicho espacio, en el período del estudio, se encontraba en una zona central de la universidad, facilitando el acceso rápido de los participantes y contaba con los accesorios necesarios para la ejecución del Programa de *Hatha-Yoga*.

### Período

Los datos fueron recolectados entre mayo y octubre de 2022.

### Población y criterios de selección

En la fase de reclutamiento del estudio, la población estuvo compuesta por estudiantes universitarios de los cursos de grado en Enfermería, Medicina, Nutrición y Psicología, siendo elegibles los 806 alumnos regularmente matriculados en este período de recolección de datos. Se excluyeron los participantes menores de 18 años, aquellos que cursaban prácticas profesionales, internado o que se encontraban en el último año o semestre de su carrera, debido a que no asistían regularmente al *campus* durante la realización del estudio. Además, se excluyeron aquellos que respondieron de manera incompleta los formularios de la investigación y, posteriormente, quienes indicaron no tener interés en participar en la intervención ofrecida.

En esta primera fase (reclutamiento), realizada entre mayo y junio de 2022, 617 estudiantes completaron los instrumentos de investigación y se convirtieron en posibles sujetos para la intervención. De este total, se excluyeron seis participantes por cuestionarios incompletos, obteniendo un total de 611 cuestionarios válidos. Posteriormente, se excluyeron 311 participantes que no manifestaron interés en participar en la intervención, totalizando así 300 participantes elegibles para ingresar al Programa de *Hatha-Yoga*.

Asimismo, el seguimiento psicológico y/o la práctica de actividades como yoga o meditación, así como el uso de medicación psicotrópica, fueron considerados criterios de exclusión; sin embargo, ninguno de los participantes cumplió con estos criterios.

### Variables del estudio

Este estudio cuasiexperimental adoptó como desenlaces primarios los indicadores emocionales evaluados antes y después de la aplicación del protocolo de intervención con *Hatha-Yoga*, los cuales incluyen: atención plena disposicional, compasión, autocompasión, síntomas depresivos, síntomas ansiosos, estrés, riesgo de suicidio actual, impulsividad y somnolencia diurna. Como desenlaces secundarios, utilizados para comparar la homogeneidad de los grupos intervención y control, se incluyeron variables sociodemográficas (sexo, edad, color de piel autodeclarado, orientación sexual, práctica religiosa, estado civil, situación laboral y condición de vivienda) y académicas (curso matriculado).

### Instrumentos de estudio

Para la caracterización muestral se utilizó un cuestionario sociodemográfico (sexo, edad, color de piel autodeclarado, orientación sexual, práctica religiosa, estado civil, situación laboral y condición de vivienda) y académico (curso), elaborado específicamente para el contexto del estudio.

Para la evaluación de los indicadores emocionales se utilizaron siete instrumentos: 1. *Mindful Attention Awareness Scale* (MAAS), escala unidimensional que evalúa los niveles de atención plena disposicional, compuesta por 15 ítems en escala Likert de 1 (casi siempre) a 6 (casi nunca), donde puntuaciones más altas reflejan mayores niveles de atención plena (α = 0,83) ^(32)^; 2. *Santa Clara Brief Compassion Scale* (SCBCS), diseñada para evaluar comportamientos de compasión hacia terceros, contiene cinco preguntas en escala Likert de 1 (nada verdadero) a 7 (muy verdadero), donde puntuaciones más altas indican mayor comportamiento compasivo (α = 0,84)^(33)^; 3. *Self-Compassion Scale* (SCS), evalúa la autocompasión y la relación con uno mismo en momentos de dificultad. Contiene 26 preguntas distribuidas en seis subescalas, con respuestas en escala Likert de 1 (casi nunca) a 5 (casi siempre), donde las puntuaciones más altas reflejan mayor nivel de autocompasión (α = 0,92)^(34)^; 4. Módulo C da *Mini International Neuropsychiatric Interview* (MINI) evalúa el riesgo de suicidio en el último mes mediante cinco preguntas dicotómicas (sí/no), cuatro de las cuales analizan comportamientos suicidas en los últimos 30 días y una durante toda la vida. El puntaje para la estratificación del riesgo varía de 0 a 33 puntos^(62)^.^(62)^; 5. *Depression, Anxiety, and Stress Scale* (DASS-21), compuesta por 21 preguntas que evalúan el estado emocional a través de tres subescalas: depresión (α= 0,92), ansiedad (α= 0,86) y estrés (α= 0,90). Utiliza una escala Likert de 0 (no se aplicó) a 3 (se aplicó mucho), con una clasificación de los síntomas en niveles normal, leve, moderado, severo y extremadamente severo^(63)^; 6. *Barratt Impulsiveness Scale* (BIS-11) mide tres aspectos del comportamiento impulsivo mediante 30 ítems en escala Likert de 1 (nunca) a 4 (muy frecuentemente), donde puntuaciones más altas indican mayor impulsividad (α = 0,62)^(64-65)^; 7. *Epworth Sleepiness Scale,* evalúa el riesgo de quedarse dormido durante actividades cotidianas. Contiene ítems graduados de 0 (ninguna probabilidad de quedarse dormido) a 3 (alta probabilidad de quedarse dormido), donde puntuaciones superiores a 10 sugieren somnolencia diurna excesiva (α = 0,83)^(66)^.

Todas estas escalas son autoadministradas y han sido adaptadas para su aplicación en adultos en el contexto brasileño, demostrando adecuadas propiedades psicométricas en sus estudios de validación^(32-34,62-64,66)^.

### Reclutamiento, muestra y asignación a la intervención

El cálculo del poder muestral para la prueba de comparación de medias en grupos independientes (GI x GC) se realizó con el *software G Power*, versión 3.1.9.7, considerando un tamaño de efecto medio (0,5), un nivel de significancia del 95% y un poder muestral del 80%, estimando una muestra de 128 sujetos a ser distribuidos en los dos grupos de testeo. Sin embargo, a pesar de los esfuerzos del equipo de investigación, no se alcanzó este tamaño muestral.

Dado que no se logró la muestra mínima prevista, se enviaron mensajes a través de *WhatsApp*® y correo electrónico para reforzar los objetivos del estudio y explicar el funcionamiento del programa de *Hatha-Yoga*, como parte del plan de reclutamiento de participantes. No obstante, a pesar de este esfuerzo, no hubo un aumento en el número de estudiantes interesados. Por lo tanto, para mitigar errores en las hipótesis, se optó por presentar medidas de tamaño de efecto en lugar de depender exclusivamente de valores p.

En la segunda fase del estudio, de junio a octubre de 2022, se formaron los grupos y se aplicó el programa de *Hatha-Yoga* durante ocho semanas. De los 300 estudiantes elegibles para esta etapa, se generó una lista numerada aleatoriamente.

Dado el riesgo de deserción, se optó por enviar invitaciones personalizadas a través de correo electrónico a cada uno de los 300 estudiantes que cumplían con los criterios de elegibilidad, con un máximo de tres intentos de contacto. La selección siguió un procedimiento secuencial: si un estudiante rechazaba la invitación, el siguiente en la lista era contactado. Un total de 264 estudiantes declinaron participar en la intervención debido a incompatibilidad horaria. Debido al bajo número de aceptaciones, no se realizó una asignación aleatoria.

En esta fase, denominada Tiempo Cero, 36 estudiantes aceptaron participar en la intervención. Los grupos fueron conformados mediante asignación simple (1:1) y organizados por un miembro del equipo de investigación (colector), siguiendo la secuencia de aceptación: el primer estudiante aceptado fue asignado al Grupo Intervención (GI), el segundo al Grupo Control (GC), y así sucesivamente hasta completar la lista de participantes.

Se optó por un GC inactivo (que recibió la intervención después de la finalización del estudio), ya que las condiciones inactivas permiten detectar los efectos reales de la intervención, además de la dificultad de encontrar una intervención placebo adecuada que no influyera en los indicadores emocionales evaluados^(67)^.

Durante las primeras cuatro semanas del programa, también denominado Tiempo Uno, cuatro participantes del GI abandonaron el estudio, lo que resultó en la siguiente distribución final: GI (n = 14) y GC (n = 18) (Figura 1).

**Figura 1 -:** Diagrama de flujo de reclutamiento y asignación de participantes basado en las directrices CONSORT (2010). Cuiabá, MT, Brasil, 2022

### Intervención

La intervención de este estudio consistió en un programa de ocho semanas de *Hatha-Yoga*. Las sesiones fueron impartidas por la primera autora, quien es instructora certificada en *Hatha-Yoga* con especialización en la metodología del Yoga experiencial, y que no participó en las etapas de reclutamiento ni en la evaluación de los participantes. El programa fue diseñado para practicantes sin experiencia previa y principiantes en yoga, consistiendo en 16 sesiones de 60 minutos de clase práctica y 15 minutos de clase teórica, totalizando 80 minutos por sesión, dos veces por semana, en el período de junio a octubre de 2022, en un espacio del campus de la institución participante.

El Programa de *Hatha-Yoga* fue sistematizado mediante un protocolo, siguiendo directrices previas^(68)^, combinando técnicas inspiradas en el *Hatha-Yoga* antiguo, premoderno y contemporáneo, tales como sonidos sagrados (*mantras*), estiramientos, elongaciones, relajación, posturas físicas (*asanas*), prácticas de limpieza (*kriyas*), gestos simbólicos (*mudras*), contracciones dirigidas (*bandhas*), ejercicios respiratorios (*pranayama*), intención compasiva (*sankalpa*), meditación y relajación, con una duración total de 60 minutos^(45,69-70)^.

Durante el desarrollo del programa, se incentivó a los estudiantes a asistir regularmente a las sesiones para garantizar la continuidad en la investigación. El Grupo Intervención (GI) se subdividió en cuatro horarios a lo largo de la semana con el objetivo de retener a los participantes y ofrecer la posibilidad de reponer sesiones en caso de ausencia, cuando fuera necesario. Los participantes del Grupo Control (GC) no recibieron la intervención; sin embargo, por razones éticas, al finalizar la investigación fueron invitados a participar en un nuevo ciclo del programa de ocho semanas de *Hatha-Yoga*.

### Recolección de datos

Los datos fueron recolectados mayoritariamente de manera presencial y en dos momentos distintos: la fase preintervención, realizada entre mayo y junio de 2022, y la fase posintervención, llevada a cabo en octubre de 2022. En la primera fase, diez recolectores previamente entrenados acudieron a las aulas y, tras obtener la autorización del profesor presente, presentaron a los estudiantes los detalles del estudio e hicieron la invitación para participar en la investigación/intervención. A los estudiantes que aceptaron responder los cuestionarios se les presentó el Consentimiento Informado (CI), con la orientación de leerlo detenidamente y firmarlo, indicando así su consentimiento e interés en participar en el estudio. El tiempo estimado para completar los cuestionarios en esta fase fue de aproximadamente 45 minutos.

En la fase posintervención, tras las ocho semanas de práctica de *Hatha-Yoga* con el GI, los mismos instrumentos fueron aplicados nuevamente a los participantes que completaron el programa, inmediatamente después de la sesión final, bajo la supervisión de los recolectores previamente entrenados. Los cuestionarios fueron almacenados en sobres sellados sin identificación de grupos. Paralelamente, los participantes del GC fueron contactados por correo electrónico por el equipo de recolección y recibieron un enlace de *Google Drive* con la versión digital de los formularios. Esta etapa fue la única realizada de manera remota.

Los datos brutos fueron digitalizados dos veces y comparados mediante el sitio *web Diffchecker*®, siendo posteriormente almacenados en los *softwares Word*®, *Excel*® y *Google Drive*.

### Análisis de datos

Los datos fueron analizados mediante técnicas descriptivas e inferenciales. En los análisis descriptivos, las variables categóricas fueron presentadas mediante frecuencias absolutas y relativas, mientras que las variables continuas fueron descritas por sus puntajes medios y respectivos desvíos estándar.

En los análisis inferenciales, las comparaciones entre proporciones fueron realizadas mediante la prueba de Chi-Cuadrado o la prueba exacta de Fisher con la extensión de Freeman-Halton, cuando era necesario. Para la comparación de medias se utilizaron pruebas t para muestras pareadas en los análisis intragrupos y pruebas t para muestras independientes en los análisis entre grupos. Los tamaños del efecto fueron calculados mediante el test g de Hedges.

Los intervalos de confianza del 95% (IC95%) fueron obtenidos a través de procedimientos de *bootstrapping* (1000 remuestreos), que generan un IC95% corregido y acelerado por sesgo (*bias-corrected and accelerated* - BCa IC95%). Esta técnica se utilizó para corregir desviaciones de normalidad en la distribución muestral y minimizar las diferencias en los tamaños de los grupos^(71)^.

Los análisis fueron realizados utilizando el *software Statistical Package for the Social Sciences* (SPSS), versión 27.

### Aspectos éticos

Este estudio fue aprobado por el Comité de Ética en Investigación con Seres Humanos, con el dictamen nº 49695621.9.0000.8124, y respetó las directrices para investigaciones en humanos establecidas en la Resolución 466/2012 del *Conselho Nacional de Saúde*, así como los principios de la Declaración de Helsinki. El estudio fue registrado en diciembre de 2021 en el *Registro Brasileiro de Ensaios Clínicos* (ReBEC) bajo el código RBR-10jgv7ky.

## Resultados

Iniciaron la intervención propuesta en este estudio 36 participantes, distribuidos equitativamente entre GI (n = 18) y GC (n = 18). Ambos grupos mostraron homogeneidad (p > 0,05) en cuanto a las características sociodemográficas, académicas y la mayoría de los indicadores emocionales evaluados en este estudio (Tabla 1), con excepción de los niveles de autocompasión, que fueron significativamente más elevados en los estudiantes del GC (p = 0,005).

El efecto de la intervención en los IE de los participantes del GI (comparación de puntajes medios pre y posintervención) se presentó en la Tabla 2.

**Tabla 1 -:** Caracterización sociodemográfica, académica e indicadores emocionales de estudiantes de grado del área de la salud según los grupos de asignación en la fase preintervención. Cuiabá, MT, Brasil, 2022

Variables	Grupo Intervención (n* = 18)	Grupo Control (n* = 18)	Valor de p
	n* (%)	n* (%)	
Sexo			0,177†
Femenino	13 (72,2%)	17 (94,4%)	
Masculino	05 (27,8%)	01 (5,6%)	
Edad‡	22,06 (±2,31)	23,72 (±7,74)	0,388 (-5,268; 1,449)§
Color de piel autodeclarado			
No blanca	16 (88,9%)	12 (66,7%)	0,228†
Branca	02 (11,1%)	06 (33,3%)	
Orientación sexual			
Heterosexual	14 (77,8%)	15 (83,3%)	1,000
Orientaciones sexuales minoritarias	04 (22,2%)	03 (16,7%)	
Práctica religiosa			
Sí	08 (44,4%)	12 (66,7%)	0,180
No	10 (55,6%)	06 (33,3%)	
Estado civil			
Con pareja	03 (16,7%)	03 (16,7%)	1,000†
Sin pareja	15 (83,3%)	15 (83,3%)	
Situación laboral			
Trabaja y estudia	05 (27,8%)	02 (11,1%)	0,402†
Solo estudia	13 (72,2%)	16 (88,9%)	
Condición de vivienda			
Vive solo(a)	03 (16,7%)	01 (5,6%)	0,603†
No vive solo(a)	15 (83,3%)	17 (94,4%)	
Curso			
Enfermería	06 (33,3%)	10 (55,6%)	0,342||
Medicina	01 (5,6%)	02 (11,1%)	
Nutrición	04 (22,2%)	01 (5,6%)	
Psicología	07 (38,9%)	05 (27,8%)	
Atención plena‡	49,21 (±12,84)	54,17 (±12,07)	0,272 (-14,208; 4,030)§
Compasión‡	25,57 (±7,42)	28,11 (±5,84)	0,287 (-6,343; 2,181)§
Autocompasión‡	68,29 (±12,46)	79,83 (±9,16)	0,005 (-17,877; -3,317)§
Depresión‡	15,71 (±6,00)	13,72 (±4,62)	0,297 (-2,574; 4,883)§
Ansiedad‡	14,71 (±4,48)	13,44 (±4,69)	0,445 (-2,062; 3,520)§
Estrés‡	17,36 (±3,71)	16,83 (±4,73)	0,736 (-2,730; 3,005)§
Riesgo de suicidio‡	0,64 (±1,15)	0,44 (±1,29)	0,655 (-0,387; 1,333)§
Impulsividad‡	68,07 (±15,27)	65,89 (±10,90)	0,640 (-6,017; 9,799)§
Somnolencia excesiva‡	10,57 (±4,07)	9,44 (±3,85)	0,430 (-2,484; 3,417)§

*n = Tamaño muestral; †Valor de p obtenido mediante la prueba exacta de Fisher; ‡Para la caracterización de variables continuas se presentan medias y desviaciones estándar; §Valor de p obtenido mediante la prueba t para muestras independientes, con IC95% obtenido por la técnica de bootstrapping con 1000 remuestreos; ||Valor de p obtenido mediante la prueba exacta de Fisher con extensión de Freeman-Halton para tablas mayores a 2x2

**Tabla 2 -:** Comparación de puntajes medios de indicadores emocionales de estudiantes asignados al grupo de intervención en los momentos pre y posintervención (n = 14). Cuiabá, Mato Grosso, Brasil, 2022

Indicadores emocionales	Grupo Intervención					
	Preintervención (n=14)	Posintervencón (n=14)	t	Valor de p	BCa IC95%*	Tamaño del efecto (g de Hedges)
	Media (de†)	Media (de†)				
Atención plena disposicional	49,21 (±12,84)	52,29 (±14,96)	1,072	0,288	-8,783; 4,699	0,21
Compasión	25,57 (±7,42)	27,93 (±5,27)	2,168	**0,050**	-4,714; -0,057	0,35
Autocompasión	68,29 (±12,46)	81,14 (±18,86)	3,165	**0,014**	-20,643; -4,084	0,77
Síntomas depresivos	15,71 (±6,00)	13,07 (±5,38)	1,459	0,171	-1,286; 6,357	0,43
Síntomas ansiosos	14,71 (±4,48)	12,50 (±4,29)	2,302	**0,032**	0,143; 4,348	0,47
Estrés	17,36 (±3,71)	16,57 (±2,34)	0,738	0,507	-1,148; 2,863	0,24
Riesgo de suicidio actual	0,64 (±1,15)	1,64 (±5,11)	0,717	0,550	-3,929; 0,643	0,30
Impulsividad	68,07 (±15,27)	66,43 (±16,48)	0,733	0,500	-1,929; 5,237	0,10
Somnolencia diurna	10,57 (±4,07)	9,21 (±3,91)	2,464	**0,045**	0,357; 2,400	0,32

*BCa IC95% = Intervalo de confianza del 95% corregido y acelerado por sesgo (*bias-corrected and accelerated*); **†**de = Desviación estándar

Se evidencia un aumento en los niveles de compasión (p<0,050) y autocompasión (p<0,014), así como una reducción de los síntomas ansiosos (p<0,032) y la somnolencia diurna (p<0,045) tras la finalización del programa de ocho semanas de Hatha-Yoga.

En el mismo período, los participantes asignados al grupo que no recibió la intervención (GC) presentaron una disminución en el indicador emocional de atención plena disposicional (p<0,013), sin evidencias de mejoras o empeoramientos en los demás IE evaluados (Tabla 3).

**Tabla 3 -:** Comparación de puntajes medios de indicadores emocionales de estudiantes asignados al grupo control en los momentos pre y postest (n = 18). Cuiabá, MT, Brasil, 2022

Indicadores emocionales	Grupo Control					
	Preteste (n=18)	Posteste (n=18)	t	Valor de p	BCa IC95%*	Tamaño del efecto (g de Hedges)
	Media (de†)	Media (de†)				
Atención plena disposicional	54,17 (±12,07)	43,72 (±12,87)	3,235	0,013	3,778; 16,919	0,80
Compasión	28,11 (±5,84)	27,00 (±8,36)	0,814	0,427	-1,426; 3,722	0,15
Autocompasión	79,83 (±9,16)	73,61 (±13,83)	0,218	0,134	-6,121; 4,255	0,52
Síntomas depresivos	13,72 (±4,62)	14,56 (±5,78)	0,753	0,440	-7,167; 0,056	0,15
Síntomas ansiosos	13,44 (±4,69)	11,94 (±4,56)	1,205	0,281	-2,778; 1,116	0,31
Estrés	16,83 (±4,73)	14,33 (±5,16)	1,676	0,138	-0,444; 3,556	0,48
Riesgo de suicidio actual	0,44 (±1,29)	3,33(±8,64)	1,401	0,279	-0,222; 5,526	0,55
Impulsividad	65,89 (±10,90)	68,11 (±11,41)	1,481	0,193	-5,608; 0,667	0,19
Somnolencia diurna	9,44 (±3,85)	10,94 (±4,54)	1,448	0,165	-3,489; 0,500	0,34

*BCa IC95% = Intervalo de confianza del 95% corregido y acelerado por sesgo (*bias-corrected and accelerated*); **†**de = Desviación estándar

Por último, la Tabla 4 compara las puntuaciones medias de los IE de los estudiantes asignados al GI y al GC en la postprueba, y en ella no se observaron diferencias estadísticamente significativas en estos parámetros. Sin embargo, se destacan las diferencias entre las puntuaciones medias de los niveles de autocompasión y atención plena disposicional, que presentaron los mayores tamaños del efecto, respectivamente g = 0,66 y g = 0,60, valores que corresponden a tamaños del efecto moderados.

**Tabla 4 -:** Comparación de puntuaciones medias de indicadores emocionales de estudiantes asignados al grupo de intervención (n = 14) y al grupo de control (n = 18) en el momento posterior a la prueba. Cuiabá, MT, Brasil, 2022

Indicadores emocionales	Postprueba					
	Grupo Intervención (n = 14)	Grupo Control (n = 18)	t	Valor de p	BCa IC95%*	Tamaño del efecto (g de Hedges)
	Media (de†)	Media (de†)				
Atención plena disposicional	52,29(±14,96)	43,72 (±12,87)	1,739	0,089	-1,280; 19,238	0,60
Compasión	27,93(±5,27)	27,00 (±8,36)	0,363	0,702	-3,850; 6,249	0,13
Autocompasión	81,14(±18,86)	73,61 (±13,83)	1,575	0,104	-2,297; 18,024	0,66
Síntomas depresivos	13,07(±5,38)	14,56 (±5,78)	0,742	0,522	-5,287; 2,696	0,26
Síntomas ansiosos	12,50(±4,29)	11,94 (±4,56)	0,351	0,464	-2,555; 3,999	0,12
Estrés	16,57 (±2,34)	14,33 (±5,16)	1,504	0,728	-0,581; 5,129	0,52
Riesgo de suicidio actual	1,64 (±5,11)	3,33(±8,64)	0,648	0,143	-6,413; 2,631	0,22
Impulsividad	66,43 (±16,48)	68,11 (±11,41)	0,341	0,735	-9,901; 6,092	0,12
Somnolencia diurna	9,21(±3,91)	10,94 (±4,54)	1,135	0,266	-4,648; 1,167	0,39

*BCa IC95% = Intervalo de confianza del 95% corregido y acelerado por sesgo (*bias-corrected and accelerated*); **†**de = Desviación estándar

## Discusión

Este estudio piloto analizó si los indicadores emocionales de los estudiantes universitarios podrían modificarse tras la realización de un programa de ocho semanas de *Hatha-Yoga*. A pesar de la ausencia de diferencias estadísticamente significativas entre el GI y el GC en el período posterior a la prueba, se evidenció, entre los estudiantes que participaron en la intervención, mejoras en algunos IE. Hallazgos congruentes con investigaciones previas que señalaban la práctica yóguica como potencialmente beneficiosa para la regulación emocional^(14)^, el equilibrio de reactividades emocionales y sentimientos/ afectos despreciativos^(53,63,72-73)^, ya que las prácticas cuerpo- mente que cultivan la compasión o la bondad amorosa contribuyen a la manutención, expansión y mejora de las emociones positivas y al fortalecimiento de comportamientos interpersonales/prosociales favorables^(53,64,74-75)^.

En este estudio, la práctica de *Hatha-Yoga* siguió un protocolo de intervención que integró técnicas psicofísicas yóguicas y un momento de acogida de los individuos para intercambios teórico-educativos sobre la ética social del yoga (*yamas*) y las observancias de sí mismo (*niyamas*), así como el compartir experiencias sobre el cuidado de sí mismo ampliado, por lo tanto, colectivo y afectivo^(23-24,76-77)^. La práctica se aplicó dos veces por semana durante ocho semanas, contribuyendo al aumento de las capacidades de compasión y autocompasión de los estudiantes de esta muestra, al mismo tiempo que disminuyó la severidad de los síntomas ansiosos y la somnolencia diurna en este grupo.

La compasión, en general, puede estimular la calma, el contacto afectivo y la afiliación^(49,78-79)^ y, a largo plazo, puede vincular propósitos de vida, sentimientos de proximidad, afectos positivos y comportamientos de conexión colectiva^(75)^. Dado que la compasión puede ser modulada, las intervenciones que la estimulan generan beneficios intrapersonales físicos y mentales, interpersonales, además de promover la cooperación y la tolerancia hacia el sufrimiento ajeno^(80-82)^, aspectos fundamentales para profesionales en formación en el área de la salud^(38)^. En consecuencia, las prácticas que propician el cultivo de la compasión pueden ser un medio y un aliado para atenuar el sufrimiento, promover un florecimiento individual y social^(48,83-86)^.

La autocompasión es la capacidad de tener conscientemente compasión por uno mismo y de amabilidad en simbiosis con la humanidad compartida y la atención plena^(87-88)^. La autocompasión no solo despierta la habilidad de afrontamiento intrapersonal, sino que también puede sensibilizar a una mayor aceptación y búsqueda de apoyo social en momentos de desafíos y sufrimiento emocional^(81,89)^.

El aumento en los niveles de autocompasión encontrado en el GI de este estudio concuerda con los resultados reportados en la literatura sobre prácticas cuerpo-mente, que demuestran que estas pueden contribuir positivamente a la modulación de la autocompasión^(87,90)^. Además, se observaron resultados similares en un estudio experimental aleatorizado con 100 estudiantes de enfermería indios, utilizando el mismo tipo y período de intervención. En dicho estudio, se verificó que el yoga fue eficaz en la mejora de la autocompasión y de la atención plena en la comparación entre los grupos (intervención y control)^(60)^. Otra investigación cuasiexperimental con estudiantes de grado en enfermería estadounidenses (n=73), cuyo objetivo era promover la autocompasión, ofreció clases semanales de yoga (una hora) durante 12 semanas y constató que el GI presentó un mayor cultivo de la amabilidad/autobondad, una de las facetas primordiales de la autocompasión, en comparación con el GC^(40)^. En este estudio no se encontraron diferencias estadísticamente significativas entre los niveles de autocompasión entre el GI y el GC en la postprueba; sin embargo, el tamaño del efecto de la diferencia de las medias fue moderado, lo que, en coherencia con los hallazgos anteriores, podría indicar un efecto importante de la práctica de *Hatha-Yoga* en este IE, que no fue evidenciado debido al pequeño poder muestral de este estudio.

El aumento de los niveles de compasión y autocompasión fortalece el proceso formativo de los estudiantes universitarios más allá de la adquisición de competencias clínicas y gerenciales, en la medida en que potencia la corresponsabilidad y la autonomía para el cuidado de sí mismo y, principalmente, motiva la formación de redes de apoyo, como amigos, compañeros, profesores, familiares y grupos de apoyo, entre otros^(13,91-92)^. La autocompasión posibilita un autocuidado ampliado, ya que agencia redes sociomateriales diversas, fortaleciendo vínculos fundamentales para profesionales directamente involucrados en el cuidado humano. Cuidar del otro implica cuidarse a sí mismo, y las redes de apoyo son elementos fundamentales en este proceso^(76-77,92)^.

En relación con los síntomas ansiosos, un IE que disminuyó en el GI después de la práctica de *Hatha-Yoga*, estos son fenómenos de orden mental y subjetivo o derivados de experiencias corporales estimuladas y aprendidas de manera distorsionada en el caótico cotidiano contemporáneo. Estos síntomas activan el sistema de alerta en el cerebro a través del eje hipotálamo-pituitaria-adrenal (HPA)^(49,93-94)^, el cual desencadena el mecanismo de “lucha y huida”. Este mecanismo, que anteriormente, en estado de homeostasis, se activaba esporádicamente, con la activación del HPA pasa a un estado de activación permanente, sobrecargando al individuo con reactividad y subjetividades despreciativas, desatención, irritabilidad e impulsividad, que en la mayoría de los casos son desproporcionadas a la realidad^(93)^. Las intervenciones con *Hatha-Yoga* aplicadas a estudiantes universitarios reafirman el potencial de la práctica para mitigar la severidad de la sintomatología ansiosa^(95)^.

En cuanto a la somnolencia diurna, aunque esta no sea una emoción en el sentido estricto, su presencia puede impactar negativamente en la calidad de vida y constituirse en un indicador de irregularidad cognitiva, conductual, mental y emocional en estudiantes universitarios^(30,96-97)^. La somnolencia diurna puede definirse como la capacidad reducida de un individuo para permanecer despierto y alerta durante las horas normales del día, lo que resulta en siestas y lapsos de somnolencia o sueño^(98-99)^.

Desde la comprensión yóguica, los sufrimientos (*duhkha-traya*) del sueño son incongruencias psicofísicas (*viksepa*) derivadas de percepciones erróneas (*bhrantidarsana*) de los estímulos internos y externos (*klesas*) por parte del individuo y de su dificultad para mantener la homeostasis entre cuerpo y mente (*manas*) ^(100)^. Independientemente de la perspectiva adoptada sobre el fenómeno, las investigaciones señalan que la somnolencia diurna puede afectar significativamente la salud mental de los universitarios en su proceso de formación académica^(101-102)^.

En un estudio de revisión sistemática y metaanálisis sobre la calidad del sueño e insomnio, se indicó que prácticas como el yoga pueden reducir la somnolencia diurna en adultos^(103)^. Otra revisión sistemática sobre aplicaciones clínicas de estudios experimentales con prácticas meditativas basadas en *mindfulness*, yoga y meditación trascendental señaló un desenlace positivo en la mejora de la calidad del sueño en jóvenes y adultos^(104)^. Las intervenciones cuerpo-mente contribuyen a la sincronización neuronal y, en consecuencia, a la mejora de las funciones globales básicas de los practicantes^(7,49)^. De esta manera, la meditación yóguica puede desempeñar un papel regulador no solo en el rendimiento cognitivo, sino también en la promoción de una restauración cuerpo-sensorio-mental integral^(104)^.

Un hallazgo importante del estudio se refiere al indicador de atención plena disposicional (APD), que, aunque se mantuvo estable en el GI en la comparación pre y postprueba, disminuyó significativamente en el GC en el mismo período. Además, se destaca que, a pesar de la ausencia de significancia estadística en el análisis entre los grupos de intervención y control, en la postprueba el tamaño del efecto encontrado fue moderado, lo que denota una diferencia importante entre los valores de las puntuaciones medias analizadas, lo que puede ser clínicamente relevante. Una posible explicación para este hecho es que el grupo de intervención podría haberse beneficiado de la práctica de *Hatha-Yoga* y, aunque no haya contribuido al aumento de los niveles de APD, pudo haber evitado su disminución, como se evidenció en el GC.

El proceso atencional comprende un ciclo en el cual el individuo alterna constantemente entre atención, divagación, conciencia de la divagación y retorno al foco^(105-106)^. En este proceso, la divagación puede atenuarse gradualmente a medida que se cultivan experiencias atencionales mediante prácticas meditativas^(49,82,107-108)^.

Aunque las publicaciones científicas sobre intervenciones con yoga para la evaluación comparativa de la APD en estudiantes universitarios aún son escasas, las evidencias disponibles sugieren mejoras en los niveles de APD tras la implementación de intervenciones yóguicas, probablemente debido a la mejora de la concentración y a la reducción de la divagación mental resultante de la práctica diaria^(90,109-110)^.

Es importante destacar que la APD está presente en el cultivo de la compasión y la autocompasión, y los estudios han señalado cada vez más correlaciones positivas entre estas y la capacidad atencional disposicional^(29,79,108)^, además de su posible contribución a la regulación emocional de síntomas ansiosos^(111)^ y a la modulación de la calidad del sueño^(41)^.

Este estudio no está exento de limitaciones. Se destaca el hecho de haber empleado la asignación simple de los participantes por orden de aceptación en la fase de constitución de los grupos control y experimental, lo cual pudo haber dificultado la medición de los efectos reales de la intervención en los indicadores emocionales evaluados. Además, se señala la falta de seguimiento (deserción) de los participantes durante la fase de aplicación del protocolo de ocho semanas de *Hatha-Yoga*, a pesar de haberse adoptado estrategias de retención, como la flexibilización de horarios para la realización de las prácticas y el envío de recordatorios semanales y mensajes de estímulo a los participantes del grupo de intervención. La deserción es un desafío particular en evaluaciones longitudinales y puede comprometer significativamente la validez e integridad de una investigación. Se sugieren nuevos estudios aleatorizados con un tamaño muestral mayor que compense las pérdidas de seguimiento y que acompañen a los estudiantes a lo largo del proceso formativo, incluyendo a estudiantes de otras áreas además de la salud.

Otra limitación importante es el tamaño muestral reclutado, que estuvo por debajo del mínimo estipulado en el cálculo de la muestra, lo que pudo haber impedido la detección de posibles efectos de la intervención sobre los indicadores emocionales. Para mitigar esta limitación, los autores optaron por presentar y discutir los resultados mediante medidas del tamaño del efecto, que indican la magnitud de los hallazgos del estudio. Por último, la adopción de un grupo control inactivo puede considerarse una limitación, por lo que se sugiere que futuros estudios incluyan un grupo control activo para permitir comparaciones de los efectos de la práctica de *Hatha-Yoga* con otras abordajes integrativos y complementarios.

Como contribución, el estudio avanza en el conocimiento científico al proponer una intervención no farmacológica como estrategia de cuidado de la salud mental de los estudiantes en el contexto universitario, con resultados significativos en la modulación de los IE, que, cuando están equilibrados, repercuten en un mejor desempeño académico y en mejores respuestas adaptativas a las demandas del entorno universitario. Además, el *Hatha-Yoga* es una práctica integrativa que fortalece a los individuos en los ámbitos personal e interpersonal/prosocial, volviéndose más segura y responsable cuando se basa en evidencias científicas.

Finalmente, se enfatiza que los resultados deben interpretarse con cautela, pues al tratarse de un estudio piloto, proporcionan insumos para la planificación de futuras investigaciones, en lugar de constituir una conclusión definitiva.

## Conclusión

Este estudio tuvo como objetivo evaluar preliminarmente los IE de estudiantes de grado en el área de la salud antes y después de la intervención con un programa de ocho semanas de *Hatha-Yoga*, resultando en un aumento significativo de la capacidad de compasión y autocompasión, así como en la reducción de síntomas ansiosos y somnolencia diurna en el grupo de intervención tras la aplicación del programa. En el grupo de control, se observó una disminución significativa en los niveles de APD. Aunque no se encontraron diferencias estadísticamente significativas en los IE entre el GI y el GC en la postprueba, se evidenció que las diferencias entre las medias de autocompasión y APD entre estos dos grupos presentaron un tamaño del efecto moderado, lo que podría indicar diferencias clínicamente relevantes.

Desde esta perspectiva, las instituciones de educación superior deben considerar el desarrollo de intervenciones no farmacológicas restaurativas, como las prácticas integrativas y complementarias en salud, en particular aquellas de tipo cuerpo-sensorio-mental, como el *Hatha-Yoga*, que incorpora la motivación del reconocimiento y cultivo integrado de la atención plena, la capacidad de compasión y la autocompasión, para apoyar el autocuidado ampliado de los estudiantes universitarios. Futuros estudios con una muestra más amplia y un seguimiento a largo plazo son necesarios para confirmar un efecto beneficioso y sus repercusiones.
